# Refractive Index‐Corrected Light‐Sheet Microscopy for Macro‐View Cardiovascular Imaging

**DOI:** 10.1002/advs.202503684

**Published:** 2025-07-13

**Authors:** Enbo Zhu, Yaran Zhang, Peng Zhao, Jae Min Cho, Zhaoqiang Wang, Yan‐Ruide Li, Jing Wang, Samuel Margolis, Shaolei Wang, Lili Yang, Alison Chu, Yuhua Zhang, Liang Gao, Tzung K. Hsiai

**Affiliations:** ^1^ Department of Bioengineering UCLA Los Angeles CA 90095 USA; ^2^ Division of Cardiology, Department of Medicine, David Geffen School of Medicine UCLA Los Angeles CA 90095 USA; ^3^ Department of Medicine Greater Los Angeles VA Healthcare System Los Angeles CA 90073 USA; ^4^ Department of Microbiology, Immunology & Molecular Genetics UCLA Los Angeles CA 90095 USA; ^5^ Eli and Edythe Broad Center of Regenerative Medicine and Stem Cell Research UCLA Los Angeles CA 90095 USA; ^6^ Jonsson Comprehensive Cancer Center, David Geffen School of Medicine UCLA Los Angeles CA 90095 USA; ^7^ Molecular Biology Institute UCLA Los Angeles CA 90095 USA; ^8^ Division of Neonatology and Developmental Biology, Department of Pediatrics, David Geffen School of Medicine UCLA Los Angeles CA 90095 USA; ^9^ Doheny Eye Institute, Department of Ophthalmology UCLA Los Angeles CA 91103 USA; ^10^ Medical Engineering California Institute of Technology Pasadena CA 91125 USA

**Keywords:** cardiovascular Imaging, large field of view, macro‐view light‐sheet fluorescent microscopy, refractive index correction

## Abstract

Light‐sheet fluorescence microscopy (LSFM) enables rapid data acquisition with minimal phototoxicity, while optical clearing reduces light scattering by matching sample and imaging medium refractive indices (RIs). Emerging clearing methods extend from cells and organoids to entire organs and organisms, prompting macro‐view LSFM microscopes with macro‐objectives for low‐magnification imaging of larger specimens at adequate resolution. In cardiovascular studies, multiple organs often require imaging, yet clearing protocols optimized for different organs alter the sample RIs inconsistently. Standard‐size dipping objectives use correction collars for RI adaptation, but macro‐objectives, owing to their large size and long working distances, are typically dry lenses lacking built‐in RI correction. An RI‐corrected (rc)‐LSFM macro‐view system addresses this limitation by accommodating a broad range of RI values. By integrating axial sweeping, multi‐view imaging, and a closed quartz chamber, the rc‐LSFM improves field of view (up to ≈8.8 mm), isotropy, spatial resolution (≈3 µm), and operational safety. It effectively visualizes microvascular networks in zebrafish embryos and post‐natal mouse retina, traces the stem cell lineage of cardiomyocytes in mouse embryos, and reveals sympathetic nerve innervation in adult mouse aorta. The rc‐LSFM macro‐view system is compatible with various optical clearing protocols for multi‐scale imaging with high isotropy and spatial resolution.

## Graphical abstract

A refractive index‐corrected light‐sheet fluorescence microscopy system enables macro‐view cardiovascular imaging across organs, adapting to refractive index variations from different optical clearing protocols. By integrating axial sweeping, multi‐view imaging, and a sealed chamber, it extends the field of view, enhances isotropy, and improves safety. This approach reveals microvascular networks, stem cell lineages, and nerve innervation for cardiovascular research.

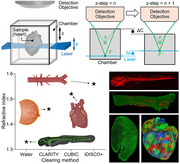

## 1 Introduction

The development of fluorescent microscopy has advanced biomedical discovery and research.^[^ ^1^, ^2^, ^3^, ^4^, ^5^ ^]^ Recent progress in light‐sheet fluorescence microscopy (LSFM) has enabled rapid and high‐resolution scanning of 3‐D samples or live animals, ^[^ ^6^, ^7^, ^8^, ^9^, ^10^, ^11^ ^]^ including *C. elegans* and zebrafish (*Danio rerio*), with minimal phototoxicity. The LSFM allows for 3‐D reconstruction of physiological architectures and functions, including the cardiac cycle,^[^ ^12^ ^]^ valvular formation,^[^ ^13^ ^]^ and micro‐circulation.^[^ ^14^ ^]^ Meanwhile, advances in optical clearing methods have extended imaging from cells and organoids to entire organs and organisms.^[^ ^15^, ^16^ ^]^ To accommodate larger specimens, macro‐view LSFM microscopes have been developed using macro objectives that offer an expanded field of view (FOV) (Table S1, Supporting Information), making them particularly suitable for whole‐organ or large‐volume imaging (Figure S1A, Supporting Information). These systems complement conventional LSFM platforms that employ standard‐size objectives with limited FOV (Table S1 and Figure S1B, Supporting Information).^[^ ^8^, ^13^, ^17^, ^18^, ^19^, ^20^ ^]^ However, adapting macro‐view LSFM for imaging across various refractive indices (RIs) remains a challenge for effectively demonstrating multi‐scale organ development, function, and interaction.

Optical tissue clearing matches the RIs of samples to the RI of the imaging medium for deep photon penetration.^[^ ^21^, ^22^ ^]^ The clearing protocol renders the tissue transparent to reduce light scattering and absorption by matching the RIs of the samples with the imaging media.^[^ ^23^ ^]^ There are three commonly utilized tissue‐clearing categories: hydrogel‐based (e.g. CLARITY),^[^ ^24^, ^25^ ^]^ hydrophobic (e.g. iDISCO+),^[^ ^26^, ^27^ ^]^ and hydrophilic (e.g. CUBIC).^[^ ^28^, ^29^ ^]^ While each clearing protocol provides unique features to dovetail to the specific tissue properties,^[^ ^21^, ^28^, ^30^, ^31^, ^32^, ^33^ ^]^ the RI of the sample is altered differently depending on the specific clearing methodology.^[^ ^34^ ^]^ Certain state‐of‐the‐art standard‐size dipping objectives, equipped with correction collars, accommodate a specific range of RIs through sophisticated optical designs that compensate for variations at the lens‐liquid interface (e.g. Nikon CFI Plan Apochromat 10XC Glyc, RI range 1.33–1.52). However, macro objectives used in macro‐view systems are impractical to implement with a dipping design due to their large size and extended working distances. These objectives are typically dry lenses with a fixed lens‐air interface, inherently lacking built‐in RI correction mechanisms.

**Figure 1:**
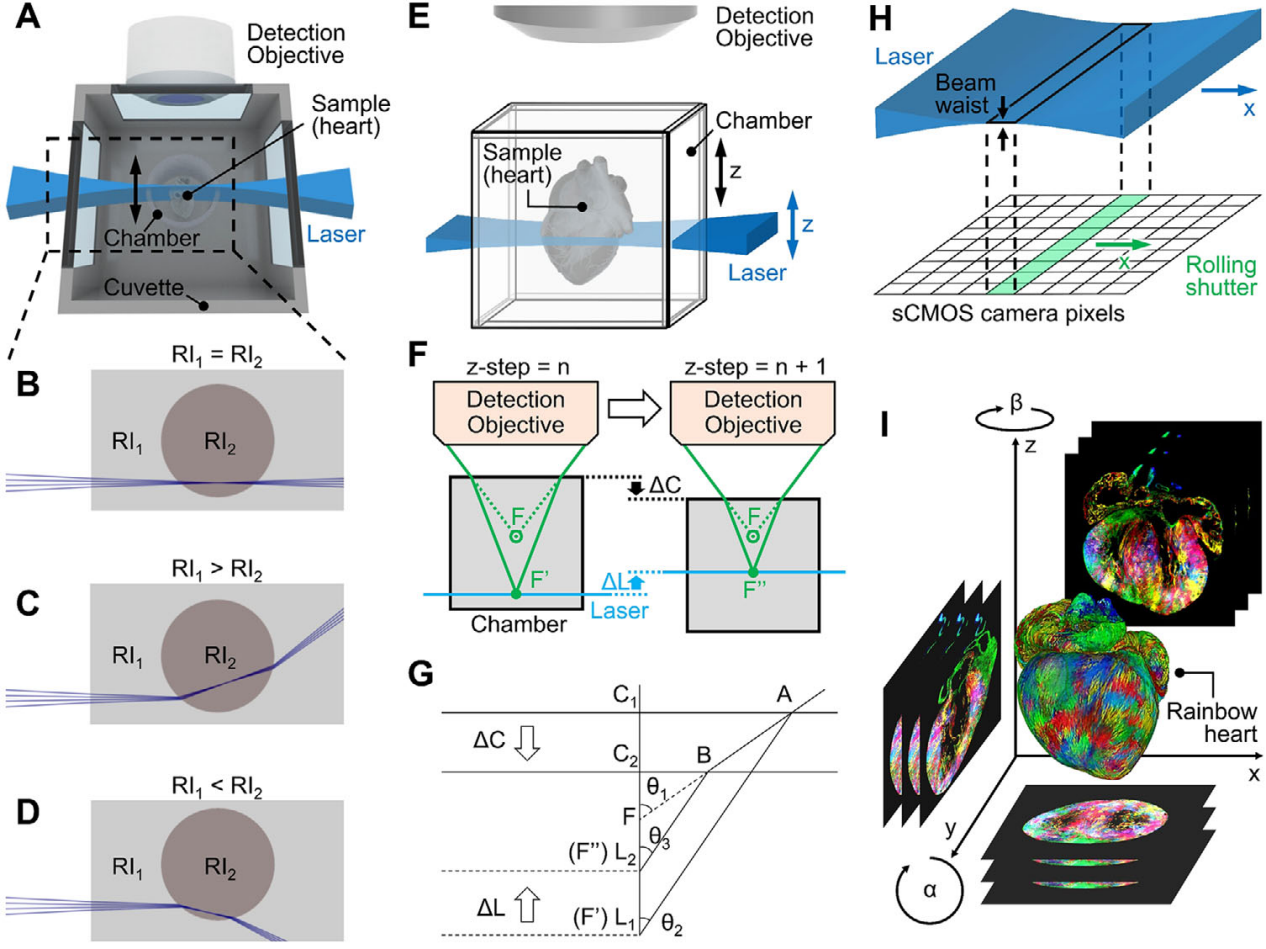
Features of the refractive index (RI)‐corrected light‐sheet fluorescence microscopy (rc‐LSFM) system. A) In a contemporary LSFM system, the chamber is placed in a large cuvette, requiring the RIs to match for each component in the cuvette. B) Matched RI_1_ (sample and imaging medium) and RI_2_ (chamber and immersion medium) enable the beam waist in (A) to scan the sample without distortion. C‐D) RI_1_ and RI_2_ mismatch results in beam refraction and distortion in (A). E) In the rc‐LSFM system, aligning the chamber displacement with the laser displacement allows for precise RI correction. F) Illumination plane is defocused (from F’ to F’’) when the chamber is displaced for the next z‐step. Simultaneous alignment of ΔC and ΔL corrects the defocus by realigning the illumination plane with F’’. ΔC: chamber displacement; ΔL: laser displacement. F represents the focus point of the detection objective in air; F’ and F’’ represent the focus points within the imaging media. G) Schematic to derive the relation between laser displacement, chamber displacement, and z‐step. H) Axial sweeping aligns the thinnest beam waist with the rolling shutter of the camera. I) Multi‐view reconstruction enhances isotropy and resolution by integrating images captured from different angles, with α representing rotation along the y‐axis and β along the z‐axis.

As a consequence, contemporary macro‐view LSFM systems have been designed for specific RIs.^[^ ^8^, ^13^, ^17^, ^18^, ^19^, ^20^ ^]^ In these systems, the sample is immersed in an imaging medium within a chamber placed in a large cuvette (**Figure** **1**A).^[^ ^8^, ^13^, ^17^, ^18^, ^19^, ^20^ ^]^ For optimal imaging quality, matching the RIs of the sample, imaging medium, chamber, and immersion medium outside the chamber is essential (Figure 1B). RI mismatches cause light to refract and reflect at interfaces, defocuses the illumination, and lead to pronounced spherical aberration (Figure 1C,D; Figures S2 and S3, Supporting Information). Consequently, LSFM systems designed for a specific RI and tissue‐clearing protocol exhibit limited adaptability to alternative clearing methods. Moreover, even within a single clearing method, fine‐tuning of the RI may be necessary for optimal performance—a flexibility that fixed‐RI systems cannot offer.^[^ ^35^ ^]^

To address these challenges, we developed the RI‐corrected (rc)‐LSFM, a macro‐view LSFM system designed to adapt to various optical clearing methods while allowing for RI fine‐tuning as needed. In contrast to conventional LSFM designs, rc‐LSFM actively compensates RI mismatch by systematically realigning the chamber and the excitation laser to maintain the beam at the focal plane of the objective lens. This compensation is based on a physical model derived from Snell's law under the paraxial approximation. As a result, rc‐LSFM achieves high‐resolution imaging across a broad RI range without requiring physical modifications to the objective or immersion medium. In addition to RI adaptability, rc‐LSFM offers several enhanced functionalities. It can determine the RIs of unknown imaging media by evaluating RI correction performance across a range of candidate values and selecting the one that yields the most accurate result. Its design enables easy sample rotation, which facilitates multi‐view reconstruction by aligning images acquired from different angles to improve overall isotropy and resolution.^[^ ^36^, ^37^, ^38^ ^]^ Moreover, the system features flexible laser movement, incorporating an axial sweeping design that synchronizes the Gaussian beam waist with the rolling shutter of the camera, achieving a large FOV without compromising resolution.^[^ ^39^, ^40^, ^41^, ^42^ ^]^ Finally, unlike conventional LSFM designs, where the imaging media are exposed, rc‐LSFM safely contains hazardous and/or evaporative media—such as dibenzyl ether (DBE)—within an enclosed sample chamber.^[^ ^43^, ^44^ ^]^

As a result, the rc‐LSFM has the capacity to uncover diverse developmental phenomena, including the ocular microvascular network in post‐natal mouse, cardiac lineage tracking of the embryonic mouse heart, nerve axon innervation to the aorta, and zebrafish microcirculation. These samples were processed using various optical clearing protocols, each with distinct RIs: the mouse ocular system was cleared by CLARITY (vasculature stained, RI = 1.45), the abdominal aorta by iDISCO+ (neurons stained, RI = 1.56), the embryonic mouse heart^[^ ^45^ ^]^ by CUBIC (cell progeny labeled, RI = 1.52), and the live transgenic *Tg(flk:mcherry)* zebrafish larva was imaged in water (vasculature labeled, RI = 1.33). Notably, the demonstrated RI range of 1.33 to 1.56 does not represent the limits of the system and could be further expanded if necessary.

Taken together, our findings demonstrate an adaptable rc‐LSFM system that integrates axial sweeping and multi‐view capability to capture a large FOV with high isotropic and spatial resolution across a wide range of RIs, thereby advancing biomedical discovery and translational research.

## 2 Results

The rc‐LSFM system was established by enabling the chamber and laser to have 6 degrees of freedom for precise alignment with the detection objective (Figure S4, Supporting Information). These degrees of freedom include long‐range adjustments (2.5 cm for translational displacement and 360° for rotational movement) that can be controlled by programming the LabVIEW. This automated control allows for precise alignments, enabling RI correction, axial sweeping, and multi‐view reconstruction. While most actuators in the system are DC servo motor actuators (Thorlabs Z825B), the actuator responsible for axial sweeping is a high‐load stepper motor (Thorlabs DRV225), capable of speeds up to 50 mm/s. With this setup, a typical 3D data acquisition involving laser axial sweeping, as demonstrated in this work, can be completed within 30 min.

In the rc‐LSFM system, the detection objective remains stationary while both the chamber and the excitation laser are simultaneously moved along the z‐axis after each frame (Figure 1E). Because there is a refractive index (RI) mismatch between the imaging medium and air, translating the chamber by itself would cause the illumination plane to defocus. The focal point of the stationary detection objective shifts (from F′ to F″) when the chamber is displaced by ∆C (Figure 1F). To counteract this defocus, the illumination plane must follow the shifted focal point of the objective. Approaches in adaptive LSFM^[^ ^46^ ^]^ involved iteratively fine‐tuning the laser position using Fast Fourier Transform (FFT) images until the optimal FFT was reached for each frame. Although effective for local laser‐position optimization, this method requires multiple FFT calculations per frame, which is time‐intensive and prone to failure in sparse or blank frames, making it unsuitable for large‐scale systematic RI corrections. In rc‐LSFM, rather than relying on iterative laser adjustments, we derive a direct physical relationship between the laser displacement (Δ*L*) related to chamber displacement (Δ*C*) (Figure 1F). This ensures rapid and robust realignment of the illumination plane with the shifted focal plane of the objective. Specifically, we establish the following relationships among the chamber displacement (Δ*C*), the laser displacement (Δ*L*), and the z‐step (Δ*S*) (Figure 1G):

(1) $$C_{2}-\;C_{1}=\Delta C$$

(2) $$L_{2}-\;L_{1}=\Delta L$$

(3) ΔS=−ΔL+ΔC

where *C*_1_ and *C*_2_ are the chamber positions before and after the shift, and *L*_1_ and *L*_2_ are the laser positions before and after the shift (Figure 1G).

From the Snell's law:

(4) $$R\;=\sin\theta_{1\;}/\sin\theta_{2}\;$$

where *R* is the RI of the imaging media.

In a macro‐view microscope (MVX‐10, Olympus) equipped with a micro objective (MVPLAPO 1X, Olympus) featuring a small numerical aperture (NA = 0.15), the paraxial approximation is justified for Equation (4), yielding the following simplified expression:

(5) $$R\;=\tan\theta_{1}/\tan\theta_{2}\;$$

where:

(6) $$\tan\;\theta_{1}=d\left(A,C_{1}\right)/d\left(F,C_{1}\right)$$

(7) $$\tan\;\theta_{2}=d\left(A,C_{1}\right)/d\left(L_{1},C_{1}\right)$$

Substituting these expressions yields:

(8) $$R=\left(L_{1}-C_{1}\right)/\left(F-C_{1}\right)$$

In addition,

(9) $$\tan\theta_{1}=d\left(B,C_{2}\right)/d\left(F,C_{2}\right)$$

(10) $$\tan\theta_{2}=\;\tan\;\theta_{3}=d\left(B,C_{2}\right)/d\left(L_{2},C_{2}\right)$$

which results in:

(11) $$R=\left(L_{2}-C_{2}\right)/\left(F-C_{2}\right)$$

where *F* is the focus point of the detection objective in the air (Figure 1F,G).

By combining Equations (1)–(3), (8), and (11), we derived the following equations:

(12) ΔS=ΔC−ΔL=ΔC·R

(13) $$\Delta L=\Delta C\left(1-R\right)$$

At a given *R*, Equations (12) and (13) demonstrate the relation between ΔS, Δ*L*, and Δ*C*.

**Figure 2:**
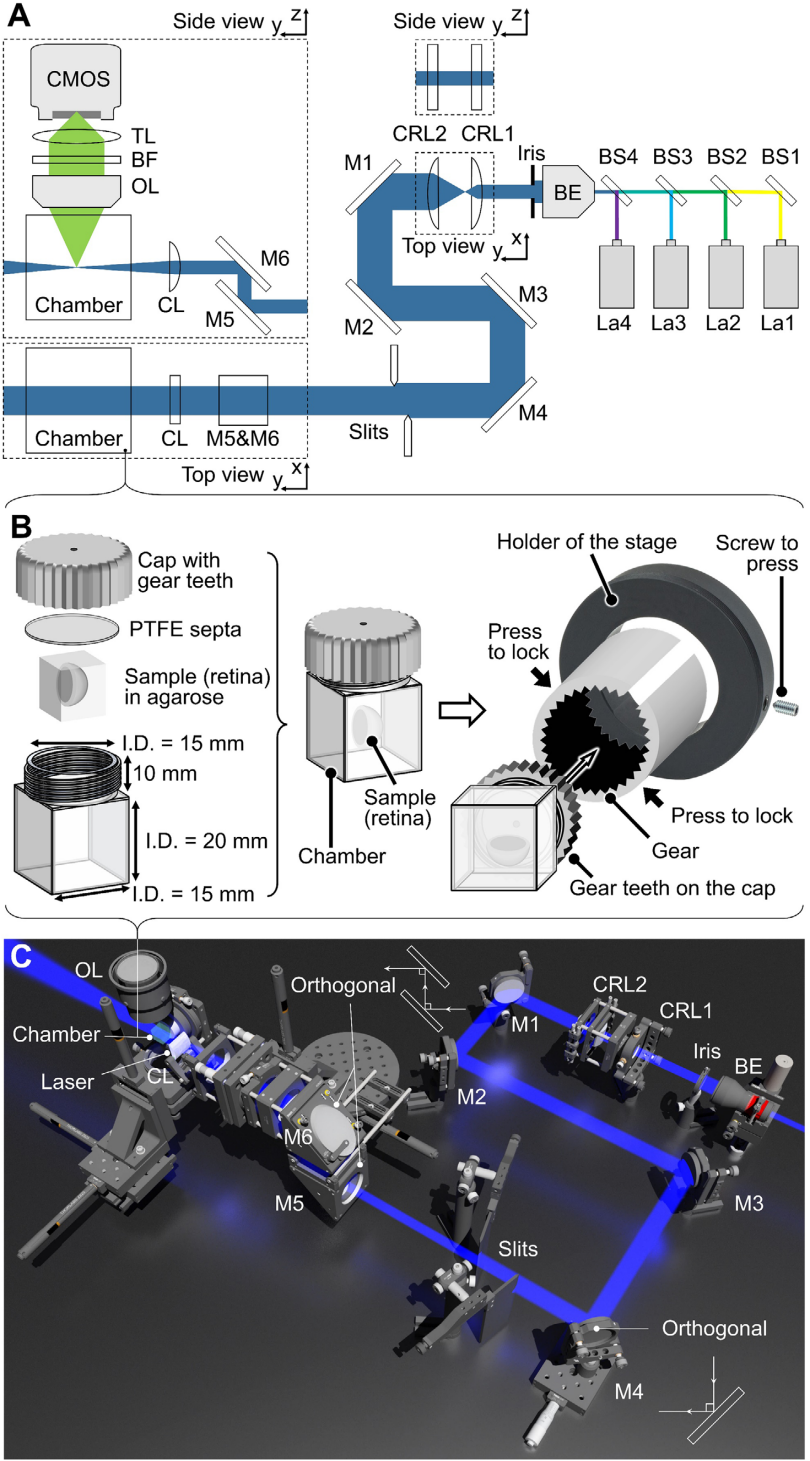
Schematic of the rc‐LSFM system. A) The optical path of the rc‐LSFM system depicts the sources of laser illumination (La1‐4) and the configuration of optical components for sample illumination and image acquisition by the CMOS camera. B) Assembly of the chamber and integration of the chamber into the rc‐LSFM system. The chamber was constructed from a single piece of quartz with a wall thickness of 1.25 mm. The gear teeth interlock the cap with the gear. C) Schematic representation of the rc‐LSFM design illustrates the optical path and various optical components. BS: beam splitter; BE: beam expander; BF: beam filter; CL: cylindrical lens; CRL: cylindrical relay lens, CMOS: Complementary metal‐oxide‐semiconductor; La: laser; M: mirror; OL: objective lens; PTFE: Polytetrafluoroethylene; TL: tube lens.

Additionally, the actuator‐controlled x‐translation of the laser aligns the beam waist with the rolling shutter of the camera, thus enabling axial sweeping (Figure 1H). The rotational freedom of the chamber further facilitates multi‐view reconstruction (Figure 1I). **Figure** **2** illustrates the optical path (Figure 2A) and configuration (Figure 2B,C; Figure S5, Supporting Information) of the rc‐LSFM system, where the orthogonal optical paths are precisely calibrated to allow independent translational control of the laser along each axis (Figure 2C). The sample is embedded in an agarose cube, which is securely anchored to the chamber by surface tension, ensuring positional stability throughout the imaging process (Figure S6, Supporting Information).

**Figure 3:**
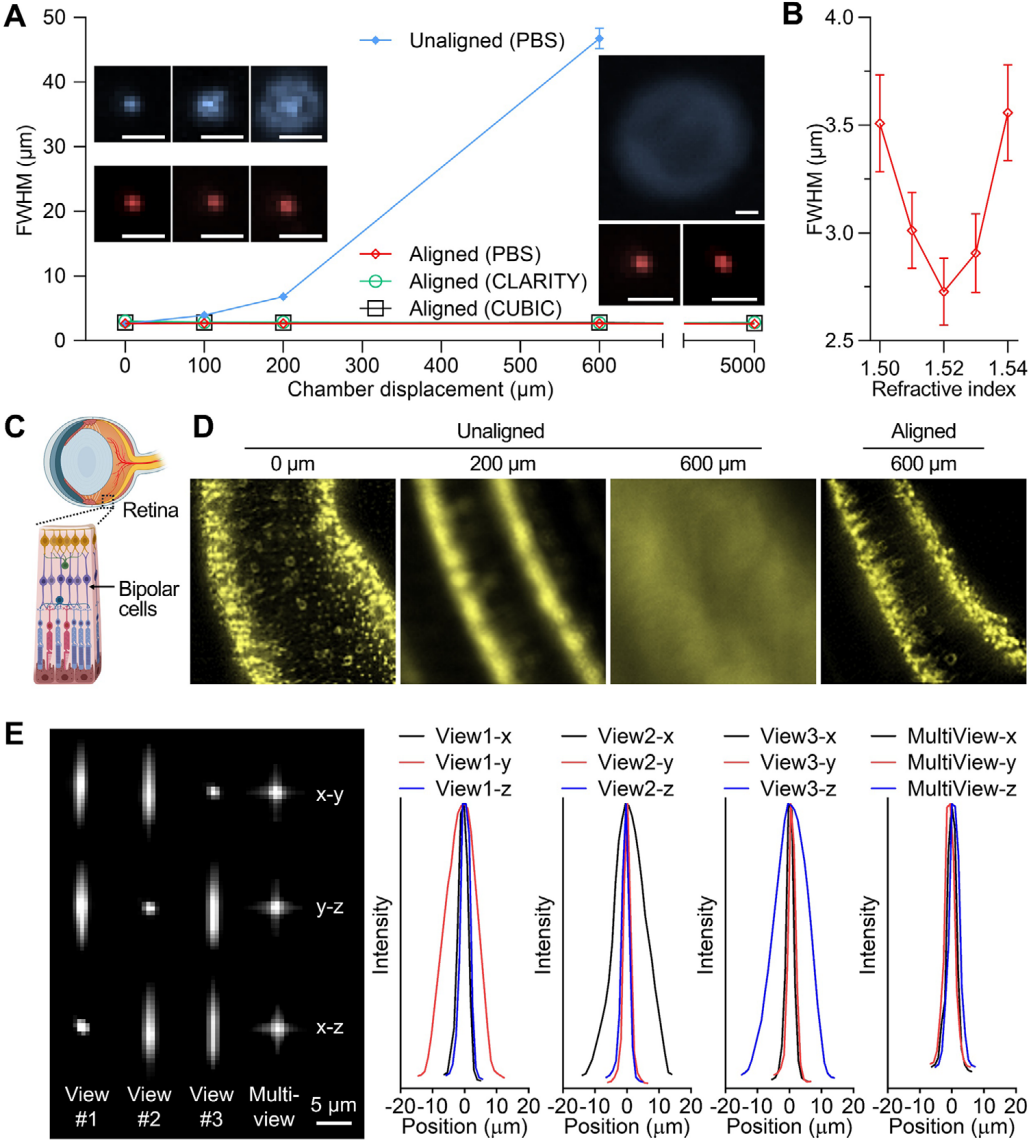
Point spread functions (PSFs) to access the rc‐LSFM imaging quality. A) Relation between chamber displacement and PSF. The unaligned configuration resulted in rapid broadening in PSF, whereas the aligned configuration maintained a high‐quality PSF among various sample clearing protocols. The RIs of the imaging media following the clearing protocols were as follows: PBS: 1.33, CLARITY: 1.46, and CUBIC: 1.52. n = 5. Scale bars: 5 µm. B) The RI of a commercial mounting solution (TCI, M3294), known to be 1.520, was determined using the full width at half maximum (FWHM) of the PSF. n = 5, chamber displacement was set to 1 cm. C) Schematic representation of bipolar cells in the retina of a mouse eye. D) Imaging of the bipolar cells labeled with anti‐(protein kinase C alpha) at various chamber displacements. Image quality degraded without laser alignment but was restored when the laser was aligned with the chamber displacement. E) PSFs from different views are compared before and after multi‐view reconstruction, demonstrating the enhanced isotropy and spatial resolution.

The point spread functions (PSFs) were evaluated under conditions of chamber displacement both with or without simultaneous laser alignment (**Figure** **3**A). When the chamber was displaced by 600 µm without laser alignment, imaging quality deteriorated, as evidenced by an increase in the PSF full width at half maximum (FWHM) from ≈2.7 µm to ≈47 µm (Figure 3A). In contrast, with simultaneous laser alignment, the PSF remained at ≈2.7 µm across a 5 mm range, independent of the RIs of the imaging media used in different clearing protocols. Spherical aberrations were further assessed through simulations at various imaging depths, wavelengths, and RIs of the media for different optical clearing methods. (Figure S7, Supporting Information) In all scenarios, the Strehl ratio exceeded 0.8, indicating minimal aberration and diffraction‐limited performance. Notably, these spherical aberrations are inherent to macro‐view LSFM in general^[^ ^8^, ^13^, ^17^, ^18^, ^19^, ^20^ ^]^ and were not exacerbated by the rc‐LSFM design. The beam waist thickness of the rc‐LSFM system was ≈10.6 µm, with a corresponding Rayleigh length of ≈644.2 µm (Figure S8, Supporting Information). Altogether, these findings demonstrate the adaptability of rc‐LSFM to varying RIs while maintaining high‐quality imaging.

In addition, the reversible relationship between the PSF and RI correction allows for determination of an unknown RI for a solution. The relationship between chamber displacement (Δ*C*) and laser displacement (Δ*L*) is governed by the RI input (Equation (13)), and any deviation from the true RI impairs the performance of the RI correction. When the rc‐LSFM system is pre‐aligned at a specific z‐height to minimize the PSF, and RI correction is subsequently applied following a chamber displacement using Equation (13), optimal PSF quality is preserved only when the correct RI is applied (Figure 3A). Therefore, by testing a range of candidate RI values, the PSF reaches its minimum at the true RI. We assessed the accuracy of RI determination using a commercial mounting solution (TCI, M3294, RI = 1.520) with our rc‐LSFM system. The results indicated that the minimum PSF corresponded to an RI of 1.52 (Figure 3B).

Further evaluation of imaging quality under chamber displacement was conducted using mouse bipolar cells (Figure 3C) stained with anti‐(protein kinase C alpha)‐(Alexa Fluor 532). Without laser alignment, a chamber displacement of 100 µm distorted the cellular structure. With laser alignment, the cellular structure remained distinctly delineated despite a chamber displacement of 600 µm (Figure 3D).

Multi‐view reconstruction was implemented in the rc‐LSFM system, and the imaging quality was evaluated using the PSF (Figure 3E). Images of 1‐µm fluorescent beads (F13082, ThermoFisher) were captured from three different orthogonal views by rotating the sample chamber along the α‐ and β‐axes (see Figure 1I). These images were then registered and reconstructed using the Multiview Reconstruction software package that accounted for content‐based local entropy.^[^ ^36^, ^37^ ^]^ Before multi‐view reconstruction, the PSF was highly anisotropic, exhibiting broader dimensions in the vertical direction. After the multi‐view reconstruction, image data from multiple angles were merged, resulting in a narrow, isotropic PSF and enhanced image quality. This methodology enables clear and isotropic visualizations of complex biological structures.

**Figure 4:**
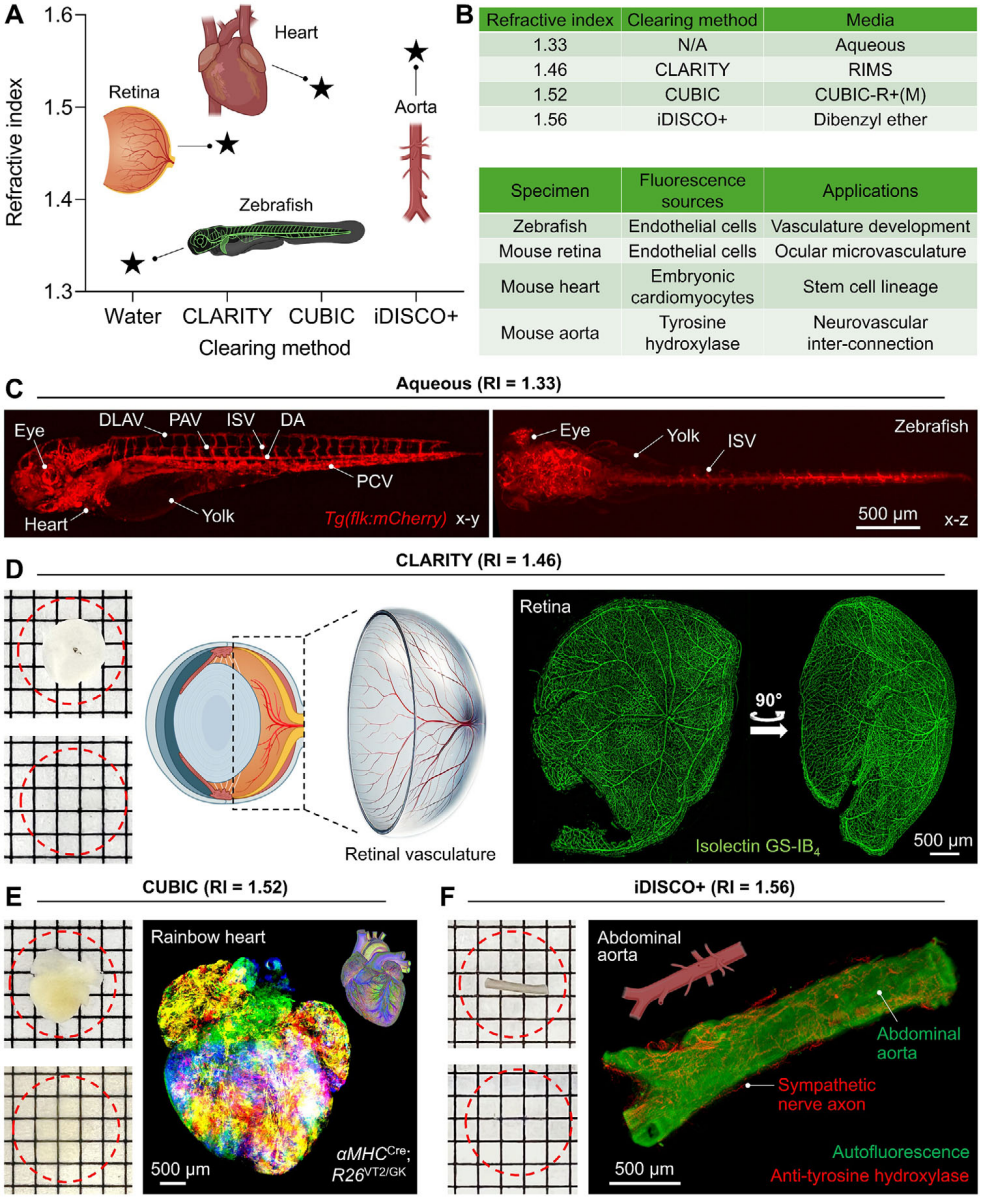
rc‐LSFM imaging embryonic zebrafish vasculature, mouse ocular microvascular plexuses, rainbow mouse heart, and sympathetic nerve axon innervation to the abdominal aorta. A‐B) Various clearing protocols suitable for different tissue properties. The imaging media exhibit distinct RIs depending on the clearing methodology. C–F) Maximum intensity projection (MIP) images of specimens processed with different clearing methods. The insets (upper) show the specimens before clearing in PBS, and the insets (lower) depict the specimens after clearing in RI‐matching imaging media. The unit length of the grid is 1.25 mm. DLAV: dorsal longitudinal anastomotic vessel; ISV: intersegmental vessels; DA: dorsal aorta; PAV: parachordal vessel; PCV: posterior (caudal) cardinal vein. Age of the specimens: C) 5 dpf; D) 12 weeks; E) Embryonic day 18.5; F) 12 weeks.

The rc‐LSFM system adapts to various RIs of a wide range of biological samples prepared by different clearing protocols^[^ ^34^ ^]^ (**Figure** **4**A,B). To demonstrate the adaptability of rc‐LSFM, we tested four commonly used clearing protocols, each associated with a specific imaging media that presents a unique RI (Figure 4A,B). Using a transgenic *Tg(flk:mcherry)* zebrafish line to study vascular development in a 1X tricaine aqueous solution (RI = 1.33),^[^ ^47^, ^48^, ^49^ ^]^ we revealed the embryonic vascular network at 5 days post fertilization (dpf) from different angles (Figure 4C). We further established the adaptability of rc‐LSFM to various optical clearing protocols; namely, CLARITY, CUBIC, and iDISCO+, to visualize the 3‐D ocular vasculature in C57BL/6 mice, the embryonic heart in αMHCCre;R26^VT2/GK^ mice, and the nerve axon innervation to the abdominal aorta in C57BL/6 mice. The samples were rendered transparent in their respective imaging media following the specific optical clearing protocols (Figure 4D–F). The mouse ocular tissue (retina) was fragile and prone to structural deformation during the clearing process (Figure S9, Supporting Information); thus, requiring a method to preserve the 3‐D structure and microvasculature. CLARITY, a hydrogel‐based method, was selected to minimize retina deformation.^[^ ^17^, ^50^ ^]^ Endothelial cells in the retinal vascular network were stained with isolectin‐B4 followed by Streptavidin‐(Alexa Fluor 488) for green fluorescence (Figure 4D). CUBIC is a superior protocol to preserve the multiple fluorescent signals, and was selected to image the αMHCCre;R26^VT2/GK^ embryonic heart, which harbors the endogenous fluorescent labels in embryonic cardiomyocytes (Figure 4E). The mouse abdominal aorta, due to its resistance to structural deformation and free of endogenous fluorescent labels, was suitable for immunolabeling and hydrophobic staining. Therefore, iDISCO+ was utilized. The aorta was stained with anti‐(tyrosine hydroxylase)‐(Alexa Fluor 594) to reveal the sympathetic nerve axons innervating the aorta (Figure 4F). Imaging time and sample size varied across specimens: the zebrafish larva (≈3.7 × 0.6 × 0.7 mm^3^) was imaged in ≈12 min; the retina (≈3.8 × 3.8 × 2.0 mm^3^) in ≈25 min; the embryonic heart (≈4.3 × 4.1 × 3.2 mm^3^) in ≈30 min; and the abdominal aorta (≈3.5 × 0.4 × 0.4 mm^3^) in ≈10 min.

## 3 Discussion

**Figure 5:**
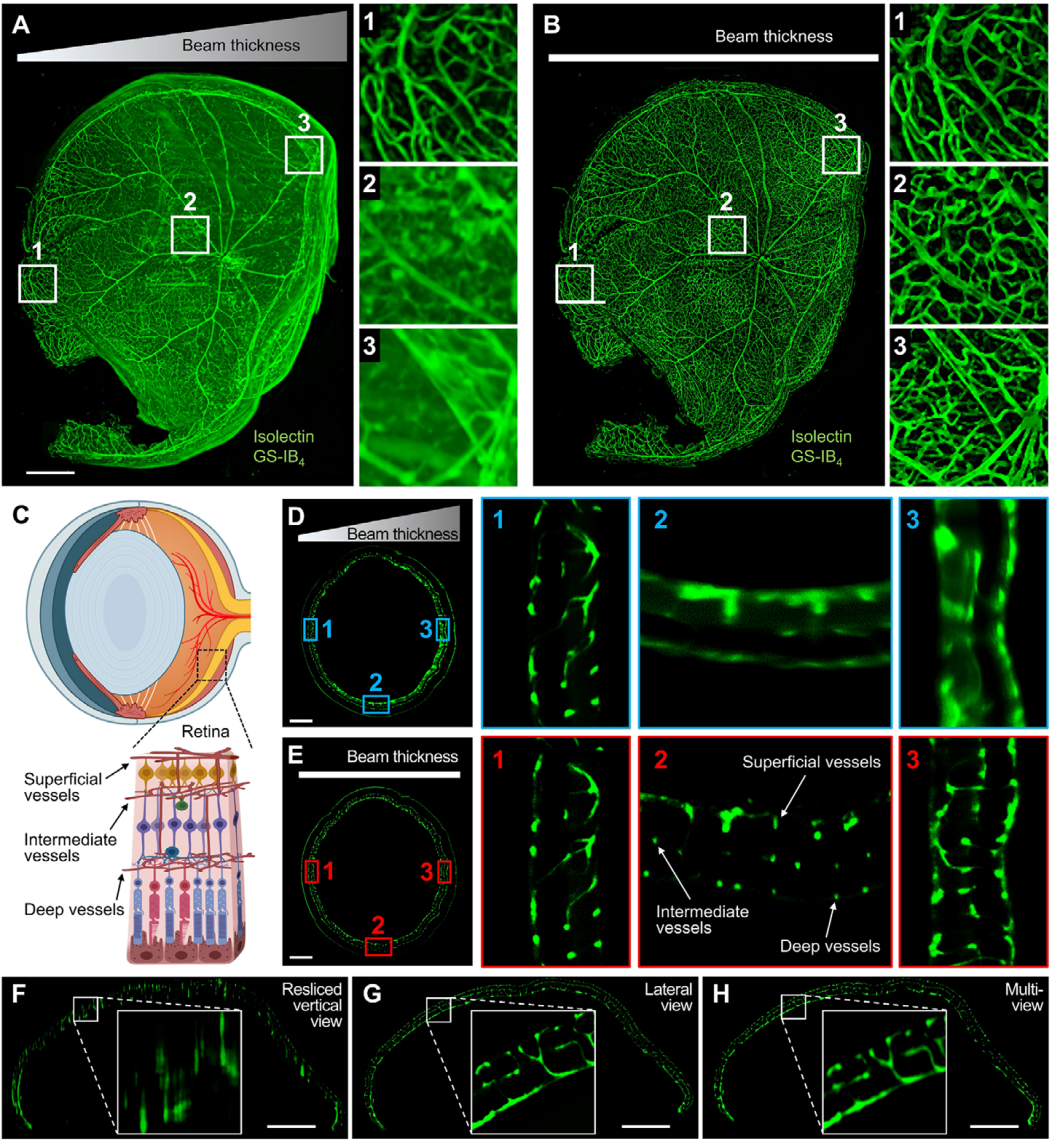
rc‐LSFM imaging the retina vasculature from a 12‐week‐old mouse. A) The maximum intensity projection (MIP) of a retina vascular network without axial sweeping. The beam waist was fixed to the left field of interest (1). B) MIP following axial sweeping. The beam waist was aligned with the camera rolling shutter to reveal high resolution in the central (2) and right fields of interest (3). C) Schematic of the retinal micro‐structure, highlighting the three layers of vascular plexuses. D) A rc‐LSFM image of the retina vasculature without axial sweeping. The beam waist was fixed to the left field of interest (1). E) The same image following axial sweeping to demonstrate the distinct three layers of vascular plexuses. F–H) Multi‐view reconstruction (axial sweeping was performed in all) enhanced isotropy and spatial resolution. F) A rc‐LSFM image acquired from a resliced vertical view. G) The same image acquired from a lateral view. H) The same image following multi‐view reconstruction. Scale bars: 500 µm.

The integration of axial sweeping to the rc‐LSFM system substantially expands the FOV without compromising resolution. After optical clearing of the retina with the CLARITY protocol,^[^ ^51^ ^]^ the maximum intensity projection (MIP) of the retinal vasculature (**Figure** **5**A,B) illustrates this advantage. When the beam waist was fixed to the left, only the left region of the sample retained high resolution (Figure 5B (1)), while the central (Figure 5A (2)) and right regions (Figure 5A (3)) became progressively blurred. In contrast, by employing axial sweeping—where the beam waist is synchronized with the camera shutter—high resolution was achieved across the left (Figure 5B (1)), central (Figure 5B (2)), and right regions (Figure 5B (3)). This improvement is also evident in individual LSFM frames. Without axial sweeping, the central and right fields were so blurred that it becomes challenging to distinguish the three vascular plexuses (superficial, intermediate, and deep) was difficult (Figure 5C,D). With axial sweeping, however, these layers become distinctly visible (Figure 5E). Note that if the beam waist is centered within the sample instead of fixed at the edge, the worst imaging decay would be comparable to what is shown in Figure 5A(2),D(2). Overall, these results demonstrate that integrating axial sweeping into the rc‐LSFM system provides a substantially larger FOV without sacrificing spatial resolution.

In addition, we incorporated multi‐view reconstruction to improve the image quality. While the same LSFM image could be acquired directly or through reslicing from an orthogonal view, the resliced vertical view (Figure 5F) exhibited lower resolution as compared to the lateral view (Figure 5G). Following multi‐view registration and reconstruction^[^ ^36^, ^37^ ^]^ (Figure 5H), the rc‐LSFM images became isotropic, achieving a high resolution approaching that of the lateral view.

Vision impairment is associated with retinal vascular dysfunction due to diabetic retinopathy,^[^ ^52^, ^53^ ^]^ glaucoma,^[^ ^54^, ^55^ ^]^ age‐related macular degeneration,^[^ ^56^, ^57^ ^]^ and retinopathy of hyperoxia in premature infants.^[^ ^17^, ^58^ ^]^ The rc‐LSFM imaged the mouse retinal microvasculature with high isotropy and resolution, revealing virtually all retinal vasculature from the arterioles to the capillaries; providing the imaging basis to further uncover neurovascular interaction in future studies.

**Figure 6:**
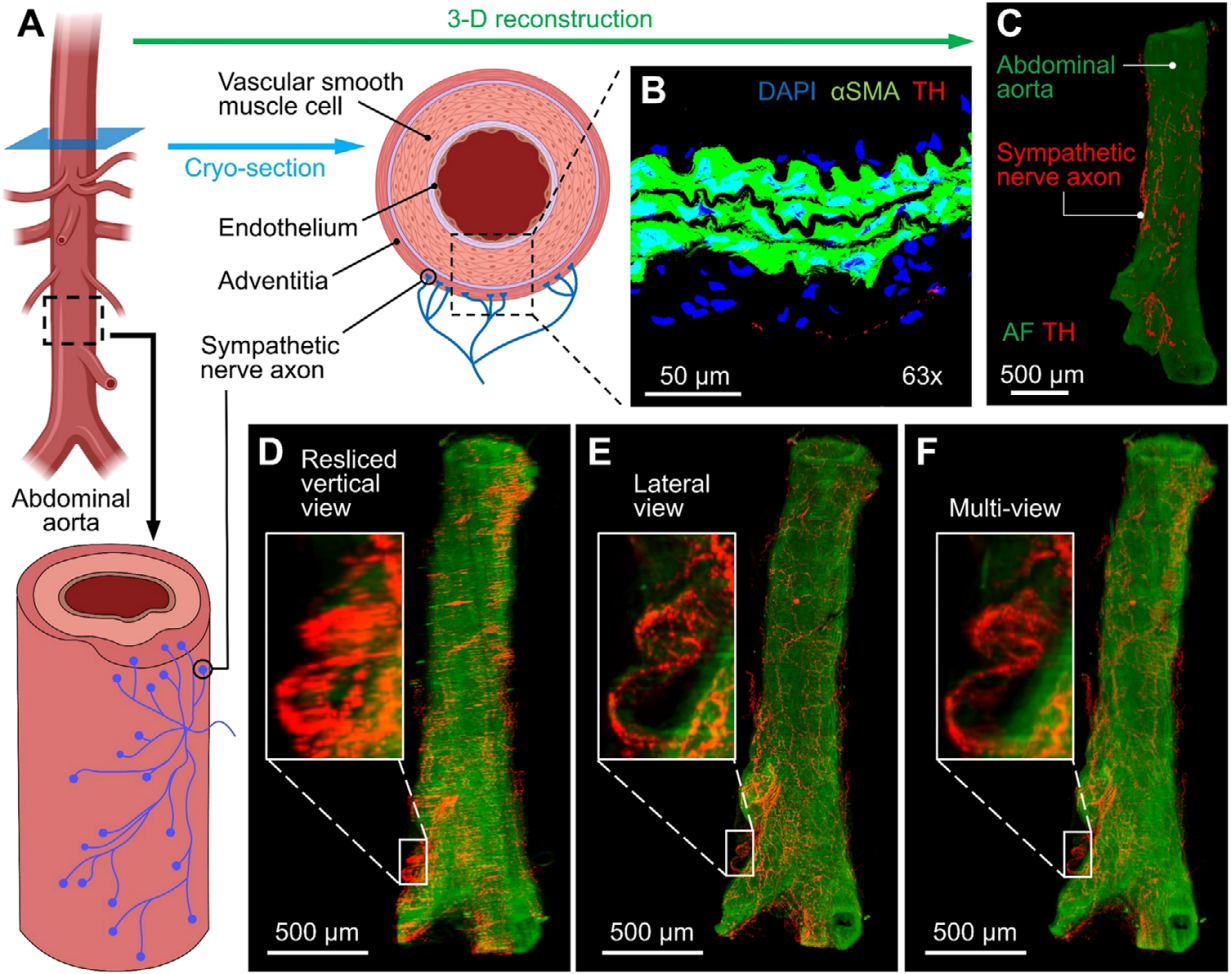
rc‐LSFM imaging sympathetic nerve axon innervating the abdominal aorta. A) Schematic representation of the abdominal aorta, highlighting the region selected for cryo‐sectioning and the sympathetic nerve axon connections to the aortic adventitia. B) Confocal image of a cryo‐sectioned abdominal aorta. αSMA: alpha‐smooth muscle actin; TH: tyrosine hydroxylase for sympathetic nerve axons. C) 3‐D reconstruction of the abdominal aorta revealing the distribution of nerve axons connecting to the aortic adventitia. AF: autofluorescence. D) MIP acquired from a resliced vertical view. E) The same MIP acquired from a lateral view. F) The same MIP acquired following multi‐view reconstruction.

The autonomic nervous system (ANS) regulates the cardiovascular system, including blood pressure, cardiac electrical conduction, and heart rates.^[^ ^59^ ^]^ Recent research has increasingly focused on the role of ANS in mediating vascular function within large arteries.^[^ ^60^ ^]^ Visualizing the scattered distribution of sympathetic nerve axons connecting to the outer layer (adventitia) of the aorta (**Figure** **6**A) has been challenging using 2‐D slices (Figure 6B). The employment of rc‐LSFM captures 3‐D nerve axons connection to the aorta (Figure 6C). We demonstrate the multi‐view reconstruction to uncover the nerve axon innervation to the mouse aorta following iDISCO+ for optical clearing^[^ ^61^ ^]^ (Figure 6D–F).

**Figure 7:**
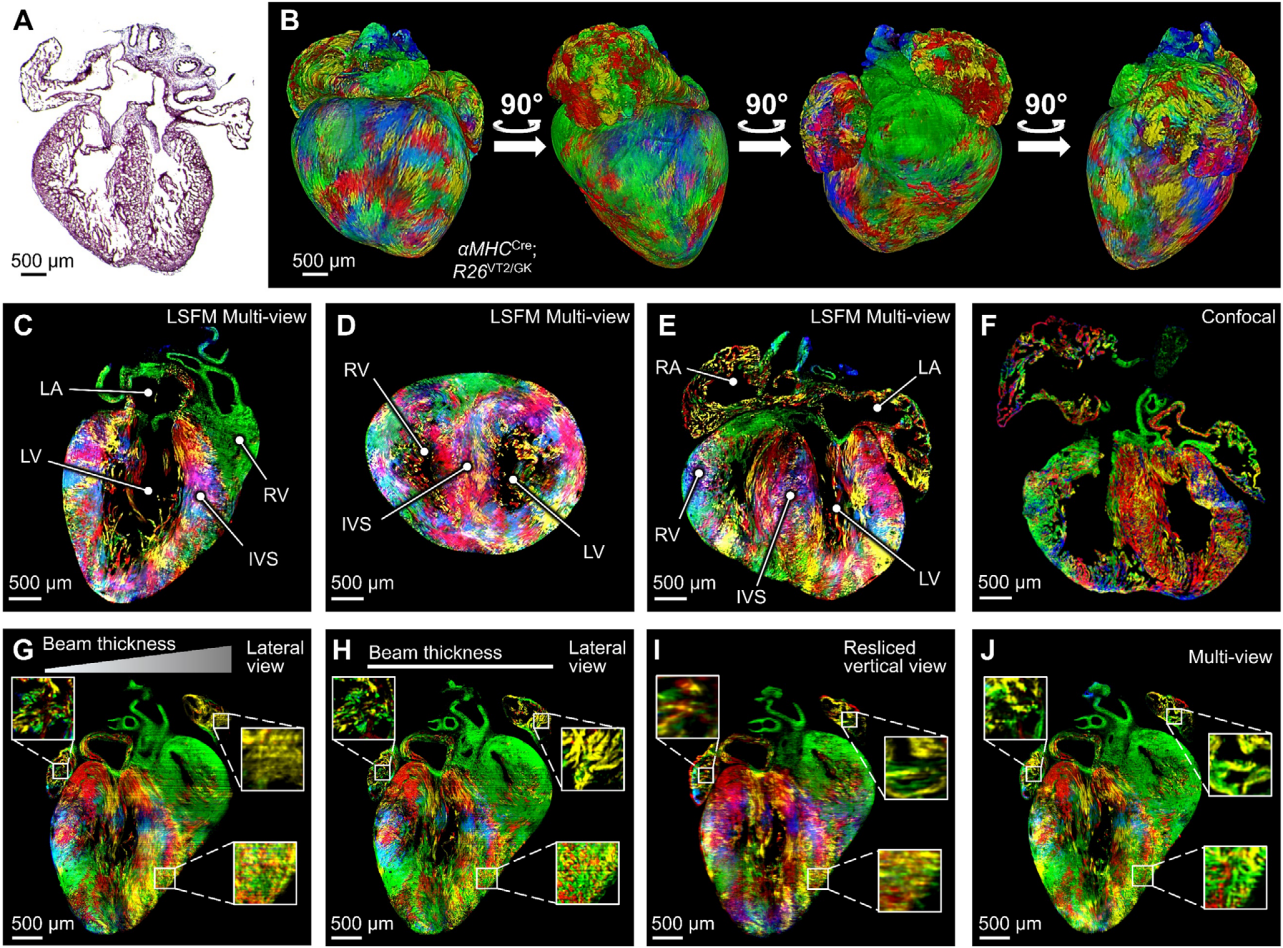
rc‐LSFM imaging αMHC^Cre^; R26^VT2/GK^ embryonic mouse heart. A) Hematoxylin and Eosin (HE) staining of an embryonic mouse heart, serving as a reference for cardiac structure. B) 3‐D reconstruction of the rainbow‐colored heart, visualized from various angles to highlight the spatial distribution of reporter expressions. C) The sagittal view image illustrates the detailed internal structures and reporter expression patterns. D) The transverse view image. E) The coronal view image. F) Confocal image of a cryo‐sectioned rainbow heart. G) The rainbow heart image acquired from a lateral view without axial sweeping. H) The same lateral view image following axial sweeping. I) The same image acquired from a resliced vertical view following axial sweeping. J) The same image following multi‐view reconstruction and axial sweeping.

The rainbow reporter system allows for the fluorescent labeling of myocardial stem cells and their progenies with unique fluorescent proteins in a stochastic manner, enabling retrospective tracking of cellular proliferation by distinct clones,^[^ ^45^, ^62^, ^63^ ^]^ otherwise challenging in the wild‐type mouse heart employing the traditional staining methods (**Figure** **7**A). The αMHC^Cre^; R26^VT2/GK^ mice were developed to demonstrate this rainbow reporter system to trace the cardiomyocyte lineages in the embryonic heart. The system utilizes Cre‐mediated recombination to mark the target cells with one of four fluorescent proteins: GFP, mCerulean, mOrange, and mCherry, all inserted into the Rosa26 (R26) locus of rainbow mice. The αMHC promoter, expressed in cardiomyocytes, induces the Cre system in mouse hearts to trace the cardiomyocyte proliferative activity (Figure S10, Supporting Information). The CUBIC protocol was employed at embryonic day 18.5 (E18.5), and rc‐LSFM was performed to reveal lineage tracing with high isotropy and resolution (Figure 7B–E). The clustering of the four fluorescent labels was visualized following the 3‐D reconstruction (Figure 7B) in the 3‐chamber sagittal, 2‐chamber transverse, and 4‐chamber coronal views (Figure 7C–E). Compared to the tiled confocal imaging of 2‐D slices, rc‐LSFM‐acquired 3‐D imaging generates a slightly lower spatial resolution but provides a comprehensive and accurate representation of 3‐D cardiac architecture (Figure 7E,F). Imaging quality was further improved via axial sweeping (Figure 7G,H) and multi‐view reconstruction (Figure 7H–J). The horizontal stripes observed in single‐view acquisitions were eliminated after multi‐view reconstruction (Figure 7G,H,J).

## 4 Conclusion

In summary, rc‐LSFM is a macro‐view system that readily adapts to the varied RIs associated with different tissue‐clearing protocols—an otherwise significant challenge for contemporary macro‐view LSFM setups. By integrating axial sweeping and multi‐view reconstruction, it provides isotropic, high‐resolution imaging across a large FOV. As a result, rc‐LSFM enables us to demonstrate 3‐D microvascular phenomena, nerve axon connections to the aorta, and cardiac lineage tracing using a single system. The configuration design is particularly well‐suited to modern macro‐view LSFM systems that employ macro objectives featuring large entrance pupil diameters and relatively small numerical apertures. In contrast, standard‐size high‐power objectives typically possess larger numerical apertures and smaller entrance pupils, leading to the breakdown of the paraxial approximation (Equation (5)) and exacerbated spherical aberrations. These optical limitations restrict the direct applicability of the rc‐LSFM design to conventional LSFM systems utilizing high‐power objectives. Overall, this macro‐view rc‐LSFM system offers a versatile solution capable of adapting to various tissue‐clearing protocols and accommodating a broad range of biological samples, thereby facilitating more comprehensive biomedical research and advancing translational applications.

## 5 Experimental Section

### Study Design

This study aimed to develop and validate a rc‐LSFM system for macro‐view cardiovascular imaging. To achieve this goal, the relationship between the chamber and laser alignment was derived to correct RI mismatches in macro‐view LSFM and demonstrated a prototype of rc‐LSFM to accommodate a broad range of RIs in various optical clearing protocols. The design also facilitate the integration of laser axial sweeping and multi‐view capability to capture a large FOV with high isotropic and spatial resolution. Imaging experiments were conducted using biological samples prepared with different clearing protocols, including CLARITY, CUBIC, and iDISCO+, to evaluate the adaptability of the rc‐LSFM system across diverse tissue types. Blinding was not implemented for data collection due to the need for precise sample handling and real‐time optimization of imaging parameters. However, objective imaging quality metrics, such as point spread function (PSF) and multi‐view reconstruction performance, were quantitatively assessed to minimize bias. Additional experimental details, including tissue preparation, optical clearing protocols, and imaging system specifications, are provided in the Supplementary Materials.

### Mouse Retina Preparation and CLARITY Tissue Clearing

A 12‐week‐old male C57BL/6J mouse, purchased from Jackson Laboratory, was anesthetized with 2–5% isoflurane The mouse was then perfused with PBS followed by 4% paraformaldehyde (PFA) in PBS. The ocular globe was extracted and then fixed in 4% PFA for 1.5 h. Next, the cornea, sclera, lens, and choroid were carefully removed, preserving the retina in its 3‐D hemispherical configuration. The retina was then immersed in 4% PFA overnight and stored in PBS at 4 °C until further processing.

Passive CLARITY clearing and immunostaining of the retina were conducted as previously reported.^[^ ^17^ ^]^ The fixed retina was incubated in a monomer solution (4% acrylamide, 0.05% bis‐acrylamide, and 0.25% VA‐044 initiator in PBS) at 4 °C overnight. Samples were then placed in a 37 °C water bath for 8 h to allow hydrogel polymerization, with a corn oil layer (Sigma, C8267) added to minimize oxygen exposure. Subsequently, the retina was incubated in a clearing solution (4% SDS and 1.25% boric acid, pH 8.5) at 37 °C to remove non‐transparent lipid contents. After clearing, the retina was rinsed in 1X PBS for 24 h to remove residual SDS, followed by incubation in the refractive index matching solution (RIMS) (40 g histodenz in 30 ml of 0.02 M PBS with 0.01% neutralized sodium azide, pH 7.5) to achieve tissue transparency.

The optically transparent retinas were placed into a blocking buffer (Perm/Block: 1X PBS + 0.3% Triton‐X + 0.2% bovine serum albumin + 5% fetal bovine serum) solution for 1 h at room temperature with gentle shaking. Primary biotinylated GS isolectin B4 (1:50, Vector lab, CA) was used to stain retinal vasculature. Following 2 days of incubation at 4 °C, retinas were washed with PBSTX (1X PBS + 0.3% Triton‐X). Streptavidin conjugated with Alexa‐488 (1:100, Invitrogen, CA) was utilized to amplify primary‐specific fluorescence. The retinas were washed with PBSTX (1X PBS + 0.3% Triton‐X) following each step.

### Preparation of the αMHC^Cre^; R26^VT2/GK^ Mouse Hearts and Optical Tissue Clearing with the CUBIC Protocol

R26^VT2/GK^ (Rainbow) mice were generously provided by Dr. Reza Ardehali. αMHC^Cre^ mice were purchased from the Jackson Laboratory. R26^VT2/GK^ (Rainbow) mice were crossed with αMHC^Cre^ mice. At embryonic day (E) 18.5, pregnant female mice were euthanized by isoflurane anesthetic overdose, followed by cervical dislocation. The uterus containing E18.5 embryos was removed through an incision at the lower abdomen and was placed in sterile PBS. Under a dissection microscope, embryos were extracted from the uterus, and their hearts were harvested. The αMHC^Cre^; R26^VT2/GK^ heart was immersed in 4% PFA overnight and stored in PBS at 4 °C until further processing.

The CUBIC clearing of the αMHC^Cre^; R26^VT2/GK^ heart was carried out following the protocol from Tokyo Chemical Industry (TCI).^[^ ^28^, ^29^ ^]^ Initially, the heart was washed in PBS with gentle shaking at room temperature twice, for 3 h each, and then stored in PBS overnight. Subsequently, the heart was incubated in a 50% CUBIC‐L (TCI, T3740) reagent solution at room temperature for 24 h. Next, the heart was transferred to CUBIC‐L reagent at 37 °C for 5 days, with the reagent being refreshed on days 1, 2, and 4. Then, the heart was washed in PBS with gentle shaking at room temperature three times, for 3 h each. It was then incubated in 50% CUBIC‐R+(M) (TCI, T3741) reagent for 24 h at room temperature, followed by a 2‐day incubation in CUBIC‐R+(M) reagent at room temperature. Finally, the heart was immersed in the mounting solution (TCI, M3294, RI = 1.520) for imaging.

### Preparation of the Mouse Abdominal Aorta with iDISCO+ Tissue Clearing

A 12‐week‐old male C57BL/6J mouse, purchased from Jackson Laboratory, was anesthetized with 2–5% isoflurane The mouse was then perfused with PBS followed by 4% PFA in PBS. The abdominal aorta was extracted, with surrounding perivascular fat carefully removed. The aorta was then fixed in 4% PFA in PBS overnight at 4 °C, then the aorta was stored in PBS at 4 °C until further processing.

Tissue clearing and immunostaining of the abdominal aorta were conducted following the iDISCO+ protocol.^[^ ^26^ ^]^ The samples were dehydrated using a methanol/H_2_O series (20%, 40%, 60%, 80%, 100%) for 1 h each. Further washing was performed with 100% methanol for 1 h. The samples were then incubated overnight with shaking in 66% DCM/33% methanol at room temperature. This was followed by two washes in 100% methanol at room temperature, and the samples were chilled at 4 °C. Bleaching occurred in chilled, fresh 5% H_2_O_2_ in methanol, overnight at 4 °C. Rehydration was performed using a methanol/H_2_O series (80%, 60%, 40%, 20%, PBS) for 1 h each at room temperature. Samples were then washed twice in PTx.2 (100 mL PBS 10X, 2 mL Triton X‐100, and water to 1 L) for 1 h each at room temperature, incubated in CytoVista Antibody Penetration Buffer (Thermofisher, V11309) at 37 °C for 2 days and blocked in CytoVista Blocking Buffer (Thermofisher, V11307) at 37 °C for 2 days. Tyrosine hydroxylase was stained with anti‐tyrosine hydroxylase antibody (Sigma, ZRB2381, clone 3M12, rabbit, 1:100), followed by washing in CytoVista Wash Buffer (V11311, diluted to 1X using PBS). Secondary staining was performed with donkey anti‐rabbit Alexa Fluor 594 antibody (Thermofisher, A‐21207, 1:200), followed by washing in CytoVista Wash Buffer. Dehydration was performed using a methanol/H_2_O series: 20%, 40%, 60%, 80%, 100%, 100%; 1 h each at room temperature, followed by a 3‐h incubation with shaking in 66% DCM / 33% methanol at room temperature. The samples were then incubated twice in 100% DCM (Sigma, 270997) and finally in DiBenzyl Ether (Sigma, 108014).

Mouse bipolar cells were imaged on retinas cleared with the iDISCO+ protocol as described above. The bipolar cells were stained with PKCα (D7E6E) Rabbit mAb (Cell Signaling, #59754, 1:100) and goat anti‐rabbit Alexa Fluor 532 antibody (Thermofisher, A‐11009, 1:100). The iDISCO+ clearing resulted in irreversible curling of the retinal structure.

### Zebrafish Line

*Tg(flk:mCherry)* embryos were obtained from natural mating at the UCLA Zebrafish Core Facility. Embryos were maintained in standard E3 medium (5 mm NaCl, 0.17 mm KCl, 0.33 mm CaCl_2_, 0.33 mm MgSO_4_ in sterile diH_2_O), cultivated at 28.5 °C, and supplemented with 1‐phenyl‐2‐thiourea (PTU, 0.003% w/v, Sigma) after 24 h post‐fertilization (hpf) to inhibit pigmentation. At 5 days post‐fertilization (dpf), embryos were anesthetized with tricaine (0.2 mg mL^−1^, Sigma Aldrich), embedded in 1% agarose (Sigma, A0576‐100G), and mounted in the LSFM chamber for imaging.

### Software

The Multiview Reconstruction software package, integrated into FIJI, was utilized for multi‐view registration and fusion. The fusion process employed content‐based weighting for the optimal results. The 3D viewer in FIJI was used to display the stacks as texture‐based volume renderings. LabVIEW, combined with the Vision Acquisition Software add‐on, controlled the entire system, ensuring alignment among the chamber, laser, and camera, and facilitating image acquisition. The geometric optical simulation was performed using Ray Optics Simulation. The aberration simulation was performed using Ansys Zemax OpticStudio, with the objective lens and tube lens inside the MVX‐10 set as paraxial lenses to evaluate aberrations only outside the system.

### Statistical Analysis

Statistical analyses were performed using Prism 10 software (GraphPad), as detailed in the corresponding figure legends. In the figures, data are presented as means ± SD. Sample sizes (n) are provided in the figure legends.

### Ethics Approval

All animal studies were performed in compliance with the IACUC protocol approved by the UCLA Office of Animal Research and in accordance with the guidelines set by the National Institutes of Health. Humane care and use of animals were observed to minimize distress and discomfort. All Zebrafish (*Danio rerio*) studies were conducted in accordance with animal protocols approved by the UCLA Institutional Animal Care and Use Committee (IACUC) (ARC#: 2015–055).

## Conflict of Interest

E.Z. and T.K.H. are inventors on patents relating to this study filed by the University of California, Los Angeles. The remaining authors declare no conflict of interest.

## Author Contributions

E.Z. and T.K.H. conceived the idea for the work. E.Z., Z.W., L.G., and T.K.H. designed the optical system, incorporating suggestions from Y.Z. (Yuhua Zhang). E.Z. constructed the experimental setups and conducted the study, with assistance from Y.Z. (Yaran Zhang), P.Z., J.M.C., Y.‐R.L., and guidance from L.Y. and A.C. for mice‐related work, as well as support from J.W. and S.G.R. for the zebrafish‐related work. E.Z. and T.K.H. wrote the manuscript, with suggestions from S.M., S.W., Y.Z. (Yuhua Zhang) and L.G. All authors had full access to all the data in the study and agreed to submit the manuscript for publication.

## Supporting information



Supporting Information

## Data Availability

The data that support the findings of this study are available from the corresponding author upon reasonable request.

## References

[1] A. D. Elliott , Curr. Protoc. Cytom. 2020, 92, 68. 10.1002/cpcy.68 PMC6961134 31876974

[2] W. Denk , J. H. Strickler , W. W. Webb , Science 1990, 248, 73. 2321027 10.1126/science.2321027

[3] W. E. Moerner , L. Kador , Phys. Rev. Lett. 1989, 62, 2535. 10040013 10.1103/PhysRevLett.62.2535

[4] E. Betzig , G. H. Patterson , R. Sougrat , O. W. Lindwasser , S. Olenych , J. S. Bonifacino , M. W. Davidson , J. Lippincott‐Schwartz , H. F. Hess , Science 2006, 313, 1642. 16902090 10.1126/science.1127344

[5] T. A. Klar , S. Jakobs , M. Dyba , A. Egner , S. W. Hell , Proc. Natl. Acad. Sci. USA 2000, 97, 8206. 10899992 10.1073/pnas.97.15.8206 PMC26924

[6] J. Huisken , J. Swoger , F. Del Bene , J. Wittbrodt , E. H. K. Stelzer , Science 2004, 305, 1007. 15310904 10.1126/science.1100035

[7] P. Fei , J. Nie , J. Lee , Y. Ding , S. Li , H. Zhang , M. Hagiwara , T. Yu , T. Segura , C.‐M. Ho , D. Zhu , T. K. Hsiai , Adv. Photonics 2019, 1, 1.

[8] Y. Chen , X. Li , D. Zhang , C. Wang , R. Feng , X. Li , Y. Wen , H. Xu , X. S. Zhang , X. Yang , Y. Chen , Y. Feng , B. Zhou , B.‐C. Chen , K. Lei , S. Cai , J.‐M. Jia , L. Gao , Cell Rep. 2020, 33, 108349. 33147464 10.1016/j.celrep.2020.108349

[9] B.‐C. Chen , W. R. Legant , K. Wang , L. Shao , D. E. Milkie , M. W. Davidson , C. Janetopoulos , X. S. Wu , J. A. Hammer , Z. Liu , B. P. English , Y. Mimori‐Kiyosue , D. P. Romero , A. T. Ritter , J. Lippincott‐Schwartz , L. Fritz‐Laylin , R. D. Mullins , D. M. Mitchell , J. N. Bembenek , A.‐C. Reymann , R. Bohme , S. W. Grill , J. T. Wang , G. Seydoux , U. S. Tulu , D. P. Kiehart , E. Betzig , Science 2014, 346, 1257998. 25342811 10.1126/science.1257998 PMC4336192

[10] G. Liu , T. Jiang , X. Li , Z. Deng , Z. Wang , H. Gong , Q. Luo , X. Yang , Optica 2023, 10, 1619.

[11] C. Mi , M. Guan , X. Zhang , L. Yang , S. Wu , Z. Yang , Z. Guo , J. Liao , J. Zhou , F. Lin , E. Ma , D. Jin , X. Yuan , Nano Lett. 2022, 22, 2793. 35324206 10.1021/acs.nanolett.1c04909

[12] P. Fei , J. Lee , R. R. S. Packard , K.‐I. Sereti , H. Xu , J. Ma , Y. Ding , H. Kang , H. Chen , K. Sung , R. Kulkarni , R. Ardehali , C.‐C. J. Kuo , X. Xu , C.‐M. Ho , T. K. Hsiai , Sci. Rep. 2016, 6, 22489. 26935567 10.1038/srep22489 PMC4776137

[13] Y. Ding , J. Lee , J. Ma , K. Sung , T. Yokota , N. Singh , M. Dooraghi , P. Abiri , Y. Wang , R. P. Kulkarni , A. Nakano , T. P. Nguyen , P. Fei , T. K. Hsiai , Sci. Rep. 2017, 7, 42209. 28165052 10.1038/srep42209 PMC5292685

[14] Z. Wang , Y. Ding , S. Satta , M. Roustaei , P. Fei , T. K. Hsiai , PLoS Comput. Biol. 2021, 17, 1009175. 10.1371/journal.pcbi.1009175 PMC8284633 34228702

[15] R. Cai , C. Pan , A. Ghasemigharagoz , M. I. Todorov , B. Förstera , S. Zhao , H. S. Bhatia , A. Parra‐Damas , L. Mrowka , D. Theodorou , M. Rempfler , A. L. R. Xavier , B. T. Kress , C. Benakis , H. Steinke , S. Liebscher , I. Bechmann , A. Liesz , B. Menze , M. Kerschensteiner , M. Nedergaard , A. Ertürk , Nat. Neurosci. 2019, 22, 317. 30598527 10.1038/s41593-018-0301-3 PMC6494982

[16] E. A. Susaki , K. Tainaka , D. Perrin , H. Yukinaga , A. Kuno , H. R. Ueda , Nat. Protoc. 2015, 10, 1709. 26448360 10.1038/nprot.2015.085

[17] C.‐C. Chang , A. Chu , S. Meyer , Y. Ding , M. M. Sun , P. Abiri , K. I. Baek , V. Gudapati , X. Ding , P. Guihard , K. I. Bostrom , S. Li , L. K. Gordon , J. J. Zheng , T. K. Hsiai , Theranostics 2021, 11, 1162. 33391527 10.7150/thno.53073 PMC7738897

[18] N. Vladimirov , F. F. Voigt , T. Naert , G. R. Araujo , R. Cai , A. M. Reuss , S. Zhao , P. Schmid , S. Hildebrand , M. Schaettin , D. Groos , J. M. Mateos , P. Bethge , T. Yamamoto , V. Aerne , A. Roebroeck , A. Ertürk , A. Aguzzi , U. Ziegler , E. Stoeckli , L. Baudis , S. S. Lienkamp , F. Helmchen , Nat. Commun. 2024, 15, 2679. 38538644 10.1038/s41467-024-46770-2 PMC10973490

[19] K. I. Baek , Y. Ding , C.‐C. Chang , M. Chang , R. R. Sevag Packard , J. J. Hsu , P. Fei , T. K. Hsiai , Prog. Biophys. Mol. Biol. 2018, 138, 105. 29752956 10.1016/j.pbiomolbio.2018.05.003 PMC6226366

[20] K. Sung , Y. Ding , J. Ma , H. Chen , V. Huang , M. Cheng , C. F. Yang , J. T. Kim , D. Eguchi , D. Di Carlo , T. K. Hsiai , A. Nakano , R. P. Kulkarni , Sci. Rep. 2016, 6, 30736. 27498769 10.1038/srep30736 PMC4976371

[21] H. R. Ueda , A. Ertürk , K. Chung , V. Gradinaru , A. Chédotal , P. Tomancak , P. J. Keller , Nat. Rev. Neurosci. 2020, 21, 61. 31896771 10.1038/s41583-019-0250-1 PMC8121164

[22] A. K. Glaser , K. W. Bishop , L. A. Barner , E. A. Susaki , S. I. Kubota , G. Gao , R. B. Serafin , P. Balaram , E. Turschak , P. R. Nicovich , H. Lai , L. A. G. Lucas , Y. Yi , E. K. Nichols , H. Huang , N. P. Reder , J. J. Wilson , R. Sivakumar , E. Shamskhou , C. R. Stoltzfus , X. Wei , A. K. Hempton , M. Pende , P. Murawala , H.‐U. Dodt , T. Imaizumi , J. Shendure , B. J. Beliveau , M. Y. Gerner , L. Xin , et al., Nat. Methods 2022, 19, 613. 35545715 10.1038/s41592-022-01468-5 PMC9214839

[23] T. Tian , Z. Yang , X. Li , J. Anat. 2021, 238, 489. 32939792 10.1111/joa.13309 PMC7812135

[24] R. Tomer , L. Ye , B. Hsueh , K. Deisseroth , Nat. Protoc. 2014, 9, 1682. 24945384 10.1038/nprot.2014.123 PMC4096681

[25] K. Chung , J. Wallace , S.‐Y. Kim , S. Kalyanasundaram , A. S. Andalman , T. J. Davidson , J. J. Mirzabekov , K. A. Zalocusky , J. Mattis , A. K. Denisin , S. Pak , H. Bernstein , C. Ramakrishnan , L. Grosenick , V. Gradinaru , K. Deisseroth , Nature 2013, 497, 332. 23575631 10.1038/nature12107 PMC4092167

[26] N. Renier , Z. Wu , D. J. Simon , J. Yang , P. Ariel , M. Tessier‐Lavigne , Cell 2014, 159, 896. 25417164 10.1016/j.cell.2014.10.010

[27] N. Renier , E. L. Adams , C. Kirst , Z. Wu , R. Azevedo , J. Kohl , A. E. Autry , L. Kadiri , K. U. Venkataraju , Y. Zhou , V. X. Wang , C. Y. Tang , O. Olsen , C. Dulac , P. Osten , M. Tessier‐Lavigne , Cell 2016, 165, 1789. 27238021 10.1016/j.cell.2016.05.007 PMC4912438

[28] K. Tainaka , T. C. Murakami , E. A. Susaki , C. Shimizu , R. Saito , K. Takahashi , A. Hayashi‐Takagi , H. Sekiya , Y. Arima , S. Nojima , M. Ikemura , T. Ushiku , Y. Shimizu , M. Murakami , K. F. Tanaka , M. Iino , H. Kasai , T. Sasaoka , K. Kobayashi , K. Miyazono , E. Morii , T. Isa , M. Fukayama , A. Kakita , H. R. Ueda , Cell Rep. 2018, 24, 2196. 30134179 10.1016/j.celrep.2018.07.056

[29] K. Tainaka , S. I. Kubota , T. Q. Suyama , E. A. Susaki , D. Perrin , M. Ukai‐Tadenuma , H. Ukai , H. R. Ueda , Cell 2014, 159, 911. 25417165 10.1016/j.cell.2014.10.034

[30] A. Greenbaum , K. Y. Chan , T. Dobreva , D. Brown , D. H. Balani , R. Boyce , H. M. Kronenberg , H. J. McBride , V. Gradinaru , Sci. Transl. Med. 2017, 9, aah6518. 10.1126/scitranslmed.aah6518 28446689

[31] E. A. Susaki , K. Tainaka , D. Perrin , F. Kishino , T. Tawara , T. M. Watanabe , C. Yokoyama , H. Onoe , M. Eguchi , S. Yamaguchi , T. Abe , H. Kiyonari , Y. Shimizu , A. Miyawaki , H. Yokota , H. R. Ueda , Cell 2014, 157, 726. 24746791 10.1016/j.cell.2014.03.042

[32] B. Yang , J. B. Treweek , R. P. Kulkarni , B. E. Deverman , C.‐K. Chen , E. Lubeck , S. Shah , L. Cai , V. Gradinaru , Cell 2014, 158, 945. 25088144 10.1016/j.cell.2014.07.017 PMC4153367

[33] V. Gradinaru , J. Treweek , K. Overton , K. Deisseroth , Annu. Rev. Biophys. 2018, 47, 355. 29792820 10.1146/annurev-biophys-070317-032905 PMC6359929

[34] F. F. Voigt , A. M. Reuss , T. Naert , S. Hildebrand , M. Schaettin , A. L. Hotz , L. Whitehead , A. Bahl , S. C. F. Neuhauss , A. Roebroeck , E. T. Stoeckli , S. S. Lienkamp , A. Aguzzi , F. Helmchen , Nat. Biotechnol. 2024, 42, 65. 36997681 10.1038/s41587-023-01717-8 PMC10791577

[35] T. Boothe , L. Hilbert , M. Heide , L. Berninger , W. B. Huttner , V. Zaburdaev , N. L. Vastenhouw , E. W. Myers , D. N. Drechsel , J. C. Rink , eLife 2017, 6, 27240. 10.7554/eLife.27240 PMC5582871 28708059

[36] S. Preibisch , S. Saalfeld , J. Schindelin , P. Tomancak , Nat. Methods 2010, 7, 418. 20508634 10.1038/nmeth0610-418

[37] S. Preibisch , F. Amat , E. Stamataki , M. Sarov , R. H. Singer , E. Myers , P. Tomancak , Nat. Methods 2014, 11, 645. 24747812 10.1038/nmeth.2929 PMC4153441

[38] Y. Wang , D. Dong , W. Yang , R. He , M. Lei , K. Shi , Photonics Res. 2024, 12, 271.

[39] K. M. Dean , T. Chakraborty , S. Daetwyler , J. Lin , G. Garrelts , O. M'Saad , H. T. Mekbib , F. F. Voigt , M. Schaettin , E. T. Stoeckli , F. Helmchen , J. Bewersdorf , R. Fiolka , Nat. Protoc. 2022, 17, 2025. 35831614 10.1038/s41596-022-00706-6 PMC10111370

[40] K. M. Dean , P. Roudot , E. S. Welf , G. Danuser , R. Fiolka , Biophys. J. 2015, 108, 2807. 26083920 10.1016/j.bpj.2015.05.013 PMC4472079

[41] L. Qiao , H. Li , S. Zhong , X. Xu , F. Su , X. Peng , D. Jin , K. Zhanghao , Adv. Photonics Nexus 2022, 2, 016001.

[42] C. Liu , C. Bai , X. Yu , S. Yan , Y. Zhou , X. Li , J. Min , Y. Yang , D. Dan , B. Yao , Opt. Express 2021, 29, 6158. 33726142 10.1364/OE.418707

[43] Y. Ding , J. Ma , A. D. Langenbacher , K. I. Baek , J. Lee , C.‐C. Chang , J. J. Hsu , R. P. Kulkarni , J. Belperio , W. Shi , S. Ranjbarvaziri , R. Ardehali , Y. Tintut , L. L. Demer , J.‐N. Chen , P. Fei , R. R. S. Packard , T. K. Hsiai , JCI Insight 2018, 3, e121396. 30135307 10.1172/jci.insight.121396 PMC6141183

[44] S. L. Logan , C. Dudley , R. P. Baker , M. J. Taormina , E. A. Hay , R. Parthasarathy , PLoS One 2018, 13, 0198705. 10.1371/journal.pone.0198705 PMC6235235 30427839

[45] K.‐I. Sereti , N. B. Nguyen , P. Kamran , P. Zhao , S. Ranjbarvaziri , S. Park , S. Sabri , J. L. Engel , K. Sung , R. P. Kulkarni , Y. Ding , T. K. Hsiai , K. Plath , J. Ernst , D. Sahoo , H. K. A. Mikkola , M. L. Iruela‐Arispe , R. Ardehali , Nat. Commun. 2018, 9, 754. 29467410 10.1038/s41467-018-02891-z PMC5821855

[46] D. P. Ryan , E. A. Gould , G. J. Seedorf , O. Masihzadeh , S. H. Abman , S. Vijayaraghavan , W. B. Macklin , D. Restrepo , D. P. Shepherd , Nat. Commun. 2017, 8, 612. 28931809 10.1038/s41467-017-00514-7 PMC5606987

[47] K. I. Baek , Y. Qian , C.‐C. Chang , R. O'Donnell , E. Soleimanian , C. Sioutas , R. Li , T. K. Hsiai , Toxics 2020, 8, 107. 33228016 10.3390/toxics8040107 PMC7711522

[48] J. Xie , E. Farage , M. Sugimoto , B. Anand‐Apte , BMC Dev. Biol. 2010, 10, 76. 20653957 10.1186/1471-213X-10-76 PMC2914679

[49] S. Gonzalez‐Ramos , J. Wang , J. M. Cho , E. Zhu , S.‐K. Park , J. G. In , S. T. Reddy , E. F. Castillo , M. J. Campen , T. K. Hsiai , Sci. Total Environ. 2023, 902, 165947. 37543337 10.1016/j.scitotenv.2023.165947 PMC10659062

[50] C. Prahst , P. Ashrafzadeh , T. Mead , A. Figueiredo , K. Chang , D. Richardson , L. Venkaraman , M. Richards , A. M. Russo , K. Harrington , M. Ouarné , A. Pena , D. F. Chen , L. Claesson‐Welsh , K.‐S. Cho , C. A. Franco , K. Bentley , eLife 2020, 9, 49779. 10.7554/eLife.49779 PMC7162655 32073398

[51] H. Du , P. Hou , W. Zhang , Q. Li , Exp. Ther. Med. 2018, 16, 1567. 30186373 10.3892/etm.2018.6374 PMC6122402

[52] N. Cheung , P. Mitchell , T. Y. Wong , Lancet 2010, 376, 124. 20580421 10.1016/S0140-6736(09)62124-3

[53] S. Sivaprasad , B. Gupta , R. Crosby‐Nwaobi , J. Evans , Surv. Ophthalmol. 2012, 57, 347. 22542913 10.1016/j.survophthal.2012.01.004

[54] R. George , S. Panda , L. Vijaya , Eye 2022, 36, 2099. 34645961 10.1038/s41433-021-01802-9 PMC9582001

[55] S. S.‐Y. Lee , D. A. Mackey , Maturitas 2022, 163, 15. 35597227 10.1016/j.maturitas.2022.05.002

[56] J. W. Miller , L. L. D'Anieri , D. Husain , J. B. Miller , D. G. Vavvas , J. Clin. Med. 2021, 10, 1124. 33800285 10.3390/jcm10051124 PMC7962647

[57] T. R. P. Taylor , M. J. Menten , D. Rueckert , S. Sivaprasad , A. J. Lotery , Eye 2024, 38, 442. 37673970 10.1038/s41433-023-02721-7 PMC10858204

[58] I. Rubinoff , R. V. Kuranov , R. Fang , Z. Ghassabi , Y. Wang , L. Beckmann , D. A. Miller , G. Wollstein , H. Ishikawa , J. S. Schuman , H. F. Zhang , Commun. Med. 2023, 3, 57. 37095177 10.1038/s43856-023-00288-8 PMC10126115

[59] C. H. Gibbons , in Handbook of Clinical Neurology (Eds.: K. H. Levin , P. Chauvel ), Vol. 160, Elsevier, Amsterdam, The Netherlands 2019, pp. 407.

[60] S. K. Mohanta , L. Peng , Y. Li , S. Lu , T. Sun , L. Carnevale , M. Perrotta , Z. Ma , B. Förstera , K. Stanic , C. Zhang , X. Zhang , P. Szczepaniak , M. Bianchini , B. R. Saeed , R. Carnevale , D. Hu , R. Nosalski , F. Pallante , M. Beer , D. Santovito , A. Ertürk , T. C. Mettenleiter , B. G. Klupp , R. T. A. Megens , S. Steffens , J. Pelisek , H.‐H. Eckstein , R. Kleemann , L. Habenicht , et al., Nature 2022, 605, 152. 35477759 10.1038/s41586-022-04673-6

[61] V. Nudell , Y. Wang , Z. Pang , N. K. Lal , M. Huang , N. Shaabani , W. Kanim , J. Teijaro , A. Maximov , L. Ye , Nat. Methods 2022, 19, 479. 35347322 10.1038/s41592-022-01427-0 PMC9337799

[62] K. Kolluri , T. Nazarian , R. Ardehali , J. Cardiovasc. Dev. Dis. 2022, 9, 141. 35621852 10.3390/jcdd9050141 PMC9145832

[63] E. Zhu , Y. Li , S. Margolis , J. Wang , K. Wang , Y. Zhang , S. Wang , J. Park , C. Zheng , L. Yang , A. Chu , Y. Zhang , L. Gao , T. K. Hsiai , VIEW 2024, 5, 20230087. 39478956 10.1002/VIW.20230087 PMC11521201

