# Supplementary material for: Refractive Index‐Corrected Light‐Sheet Microscopy for Macro‐View Cardiovascular Imaging
Source: Adv Sci (Weinh). 2025 Jul 13;12(38):e03684. doi: 10.1002/advs.202503684 (PMC12520573; doi:10.1002/advs.202503684)
Supplement: Supplementary file 1 — Supporting Information [file ADVS-12-e03684-s001.pdf]

ADVANCED SCIENCE
Open Access

Supporting Information

for *Adv. Sci.*, DOI 10.1002/advs.202503684

Refractive Index-Corrected Light-Sheet Microscopy for Macro-View Cardiovascular Imaging

*Enbo Zhu, Yaran Zhang, Peng Zhao, Jae Min Cho, Zhaoqiang Wang, Yan-Ruide Li, Jing Wang, Samuel Margolis, Shaolei Wang, Lili Yang, Alison Chu, Yuhua Zhang, Liang Gao and Tzung K. Hsiai**

Supporting Information

Refractive Index-Corrected Light-sheet Microscopy for Macro-View Cardiovascular Imaging

Enbo Zhu, Yaran Zhang, Peng Zhao, Jae Min Cho, Zhaoqiang Wang, Yan-Ruide Li, Jing Wang, Samuel Margolis, Shaolei Wang, Lili Yang, Alison Chu, Yuhua Zhang, Liang Gao, Tzung K. Hsiai*

*Correspondence email: THsiai@mednet.ucla.edu

Supplementary Tables

Table S1: Comparison Between Standard-Size and Macro Objectives

Numerical Aperture	Objective size	Objective Name[1]	Working Distance	Field of View[2]
0.15	Standard-size	MPLFLN 5X	20 mm	2.66 mm
	Macro	MVPLAPO 0.63X	87 mm	21.11 mm
0.25	Standard-size	PLN 10X	10.6 mm	1.33 mm
	Macro	MVPLAPO 1X	65 mm	13.31 mm
0.5	Standard-size	LMPLFLN 50X	10.6 mm	0.27 mm
	Macro	MVPLAPO 2XC	20 mm	6.66 mm

[1]All objectives listed are commercially available from Olympus Evident.

[2]The field of view values were calculated based on sensor dimensions without accounting for any intermediate optical components; a standard sensor size of 13.312 mm was used in all calculations for consistency.

Supplementary Figures

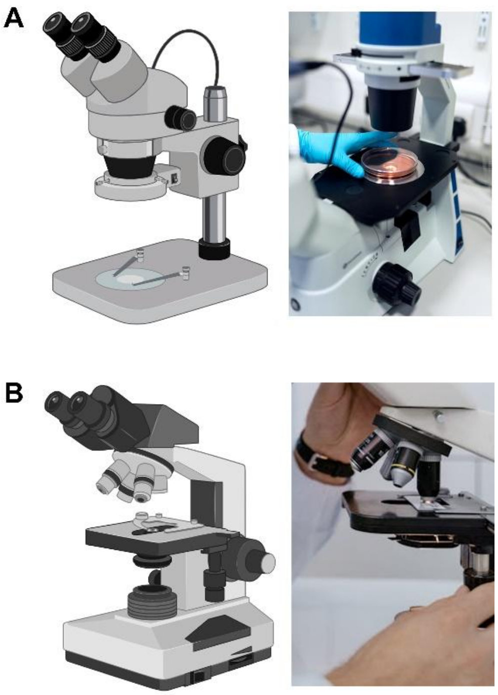

Figure S1. Schematics and comparison between a macro-view microscope and a conventional microscope. A) A macro-view microscope equipped with a macro objective enables low-magnification imaging with a large field of view while maintaining satisfactory resolution. The large size of the macro objective allows for an extended working distance; however, it is impractical for immersion in liquid due to its bulky design. B) A conventional microscope, utilizing one or multiple standard-size high-power objectives, provides high-magnification imaging with high resolution. However, the working distance and field of view are inherently limited. Standard-size objectives can be engineered for immersion in liquids, incorporating complex optical designs such as a correction collar to compensate for refractive index (RI) variations.

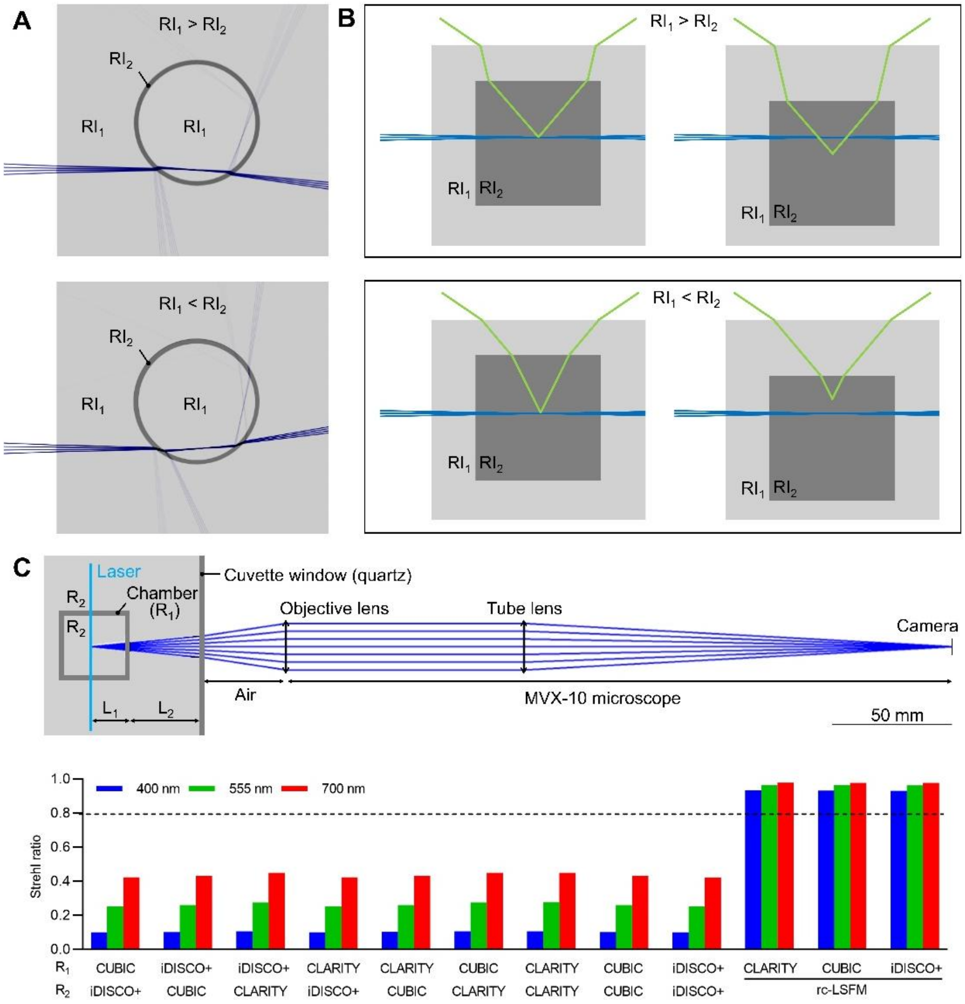

Figure S2. Imaging quality degradation in contemporary macro-view LSFM systems due to RI mismatch. A) RI mismatch from the chamber induces light reflection, reducing image quality. B) Although a square chamber in the cuvette eliminates light distortion at the interfaces, RI mismatch during sample movement causes defocusing of the illumination plane. C) Contemporary macro-view LSFM systems exhibit stronger spherical aberration compared to rc-LSFM. A higher Strehl ratio indicates lower spherical aberration, and Strehl ratios above 0.8 reflect minimal aberrations and diffraction-limited performance. ZEMAX setup: The MVPLAPO 1X objective has a 65 mm working distance (NA = 0.15). L_1: 9 mm; L_2: 50 mm. The media depth in rc-LSFM is also 9 mm.

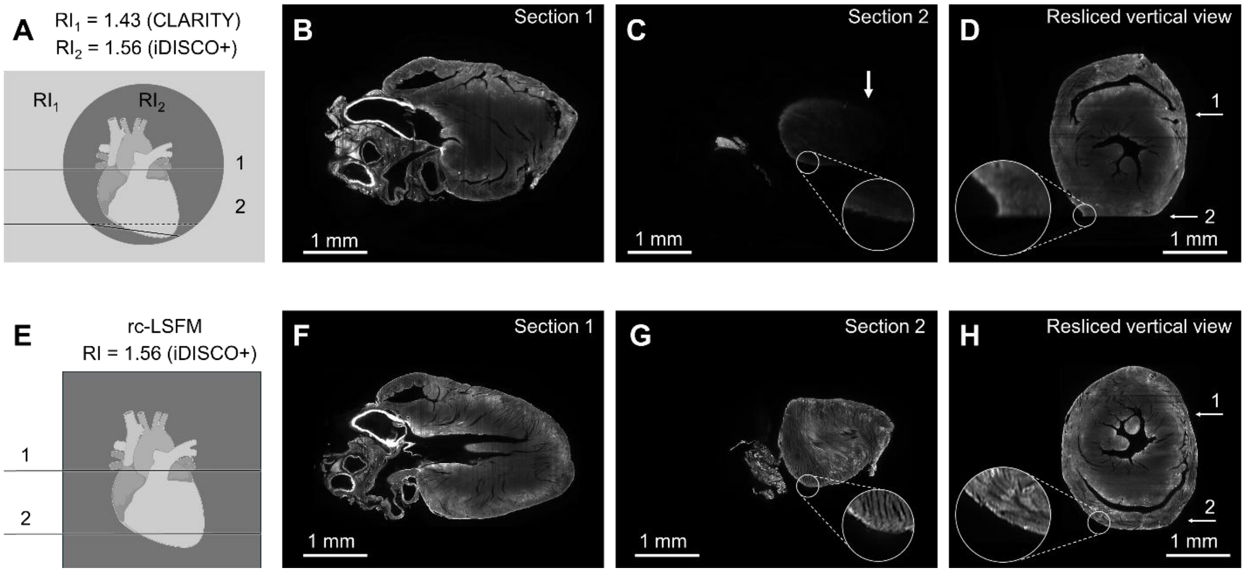

Figure S3. Imaging performance comparison of an iDISCO+ cleared 12-month-old mouse heart (~8.8 × 5.2 × 5.1 mm^3) using a CLARITY-optimized system versus the rc-LSFM system. Autofluorescence was excited at 589 nm. A) Schematic of RI mismatch during imaging with a CLARITY-optimized system. Two optical sections were selected for analysis. B) Optical image of section 1 in A. The image appears sharp and clear, as light distortion is minimal at this depth. C) Optical image of section 2 in A. Significant light distortion occurs at the RI_1-RI_2 interface, resulting in defocus and signal attenuation, especially in the region indicated by the arrow. D) Resliced vertical view reveals signal loss at the lower region beneath section 2. Although signals are present in the circled region, the structure appears distorted. E) Schematic of the rc-LSFM setup and selected sections. F) and G) Optical images of sections 1 and 2 in E. Both images remain sharp and clear, with no evident defocus or signal loss. H) Resliced vertical view confirms preservation of signals throughout the depth, including the lower region below section 2. No structural distortion, as seen in panel D, is observed.

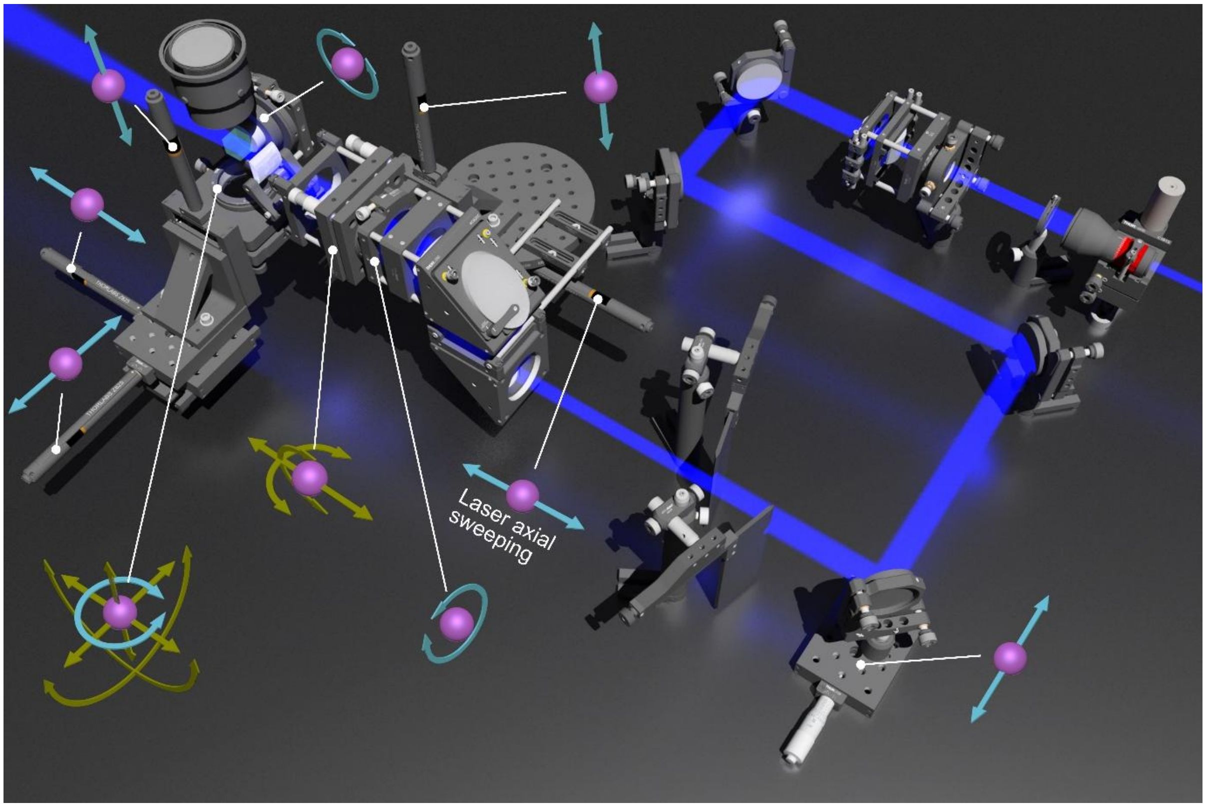

Figure S4. Degrees of freedom in the rc-LSFM system. The rc-LSFM system is designed with 6 degrees of freedom for both the chamber and laser. These degrees of freedom are categorized into local (green) and long-range (cyan) adjustments. The chamber supports three translational degrees of freedom (up to 2.5 cm) and two rotational degrees of freedom (360°) for long-range adjustments. The laser offers three translational degrees of freedom (up to 2.5 cm) and one rotational degree of freedom (360°) for long-range adjustments. All actuators are DC servo motor actuators (Thorlabs Z825B), except for the one for laser axial sweeping, which utilizes a high-load stepper motor actuator (Thorlabs DRV225, 50 mm/s)

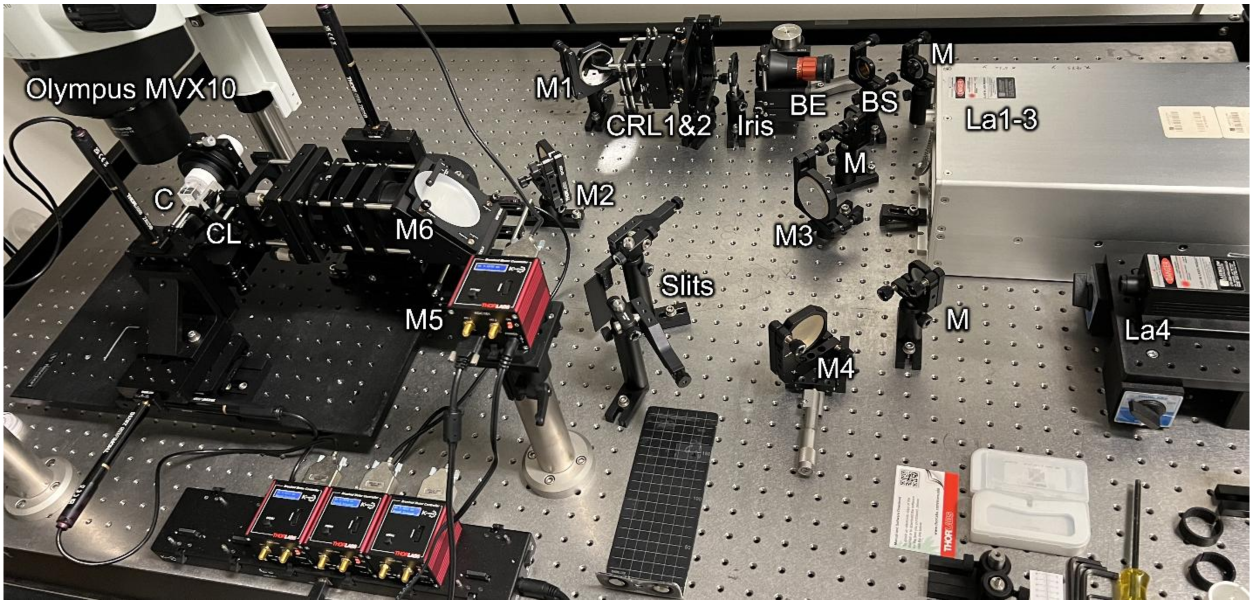

Figure S5. Optical components in the rc-LSFM system. La: laser; M: mirror; BS: beam splitter; BE: beam expander; CRL: cylindrical relay lens; CL: cylindrical lens; C: chamber.

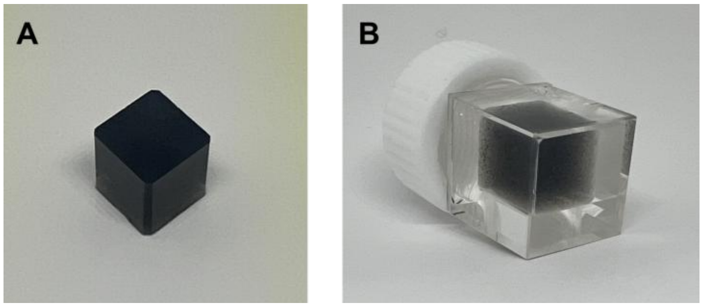

Figure S6. Mounting agarose in the imaging chamber. A) A 1% agarose cube with trace amounts of carbon powder to aid visualization. In actual experiments, the agarose is transparent and contains the embedded sample. B) The agarose cube placed in the chamber with three-facet contact and immersed in a CUBIC RI-matching mounting medium (TCI, M3294, RI = 1.520), representing the standard configuration used in actual experiments to ensure stability. Surface tension, along with multi-surface contact, prevents slippage or detachment even when the chamber is rotated or inverted.

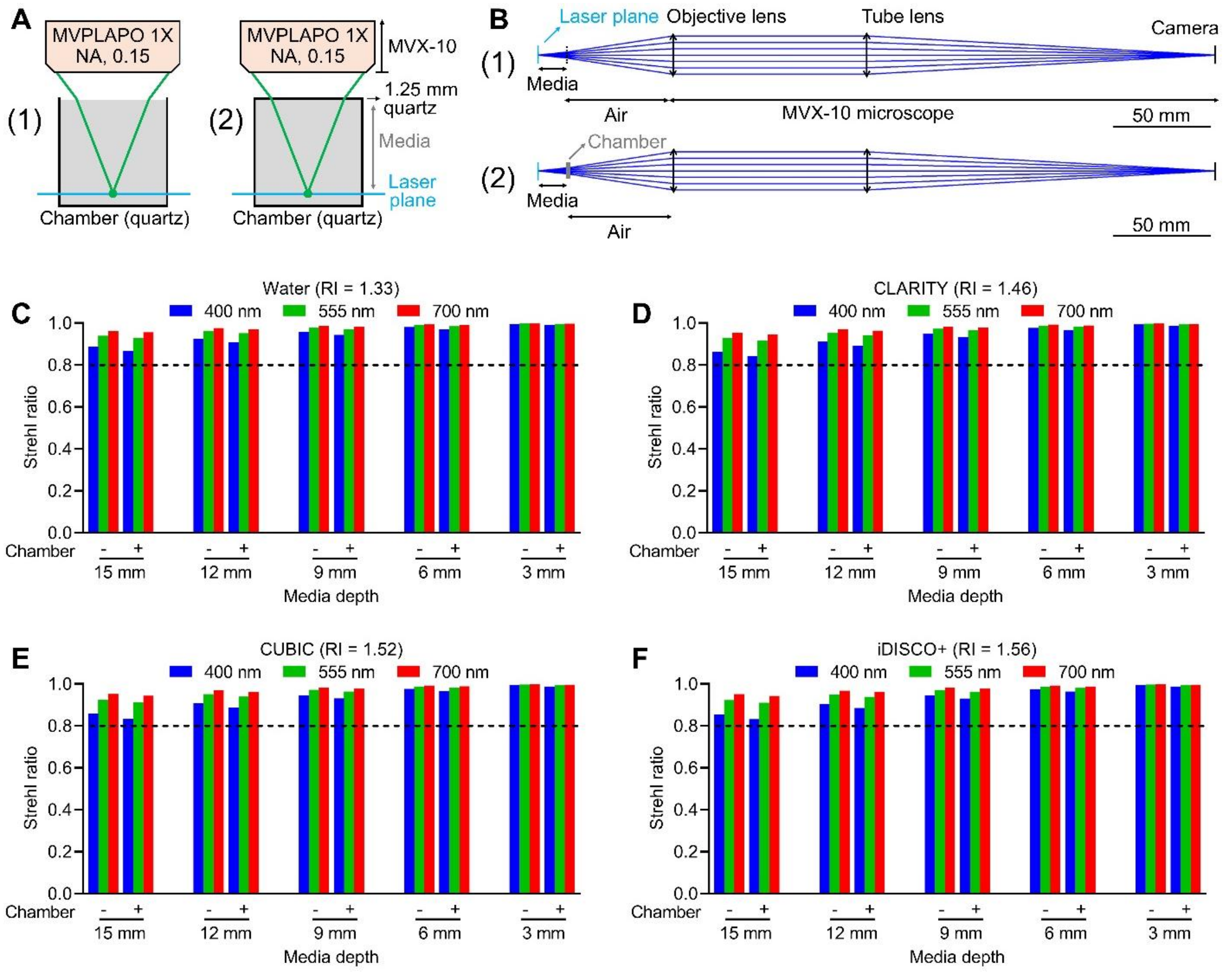

Figure S7. Evaluation of Spherical Aberration in the rc-LSFM System. A) Schematics of two simulation configurations. Spherical aberration can arise from refractive index mismatches in the immersion media and imaging chamber. Without a top chamber, multi-view imaging is disabled. B) ZEMAX simulation setup. The MVX-10 objective (MVPLAPO 1X) has a 65 mm working distance, and the MVX-10 tube lens (MVX-TLU) has a 180 mm focal length. C-F) Strehl ratio evaluations across varying imaging depths, wavelengths, and RI-matching media for different optical clearing methods. Strehl ratios exceed 0.8 in all cases, indicating minimal aberrations and diffraction-limited performance.

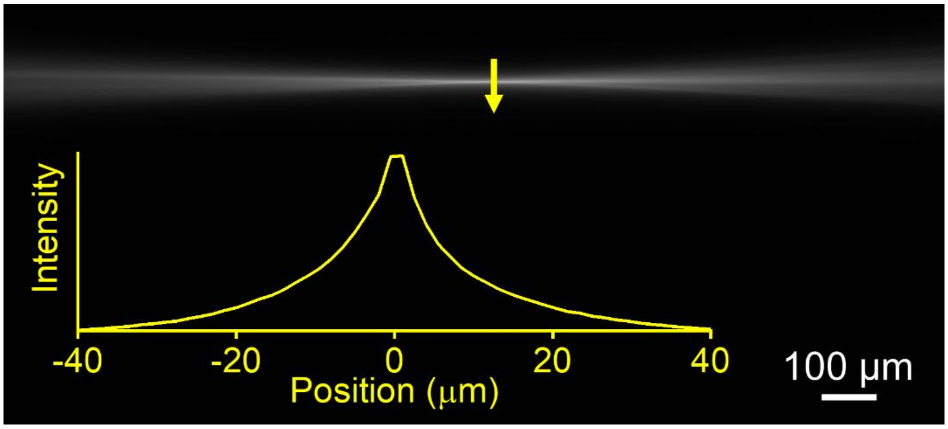

Figure S8. The full width at half maximum (FWHM) of the focused beam is measured as ~12.5 μm. The beam waist radius ω_0 is estimated as FWHM/1.177 = ~10.6 μm. The corresponding Rayleigh length z_R is calculated using the equation $z_R = \pi \cdot \omega_0^2/\lambda$, yielding z_R= ~644.2 μm for a wavelength λ=550 nm.

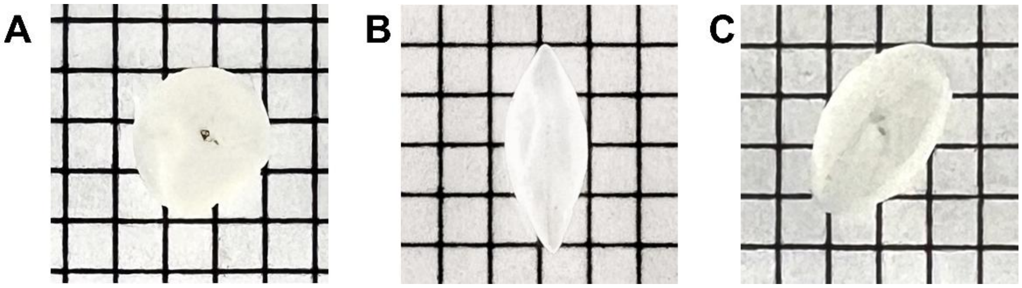

Figure S9. Structural deformation of mouse retina during non-hydrogel-based clearing processes. A) Freshly dissected and fixed mouse retina in PBS prior to the optical clearing procedure. B) Following the iDISCO+ procedure that entails dehydration and rehydration, the mouse retina in PBS developed irreversible curling. C) Following the CUBIC procedure that entails delipidation, the mouse retina in PBS also developed irreversible curling.

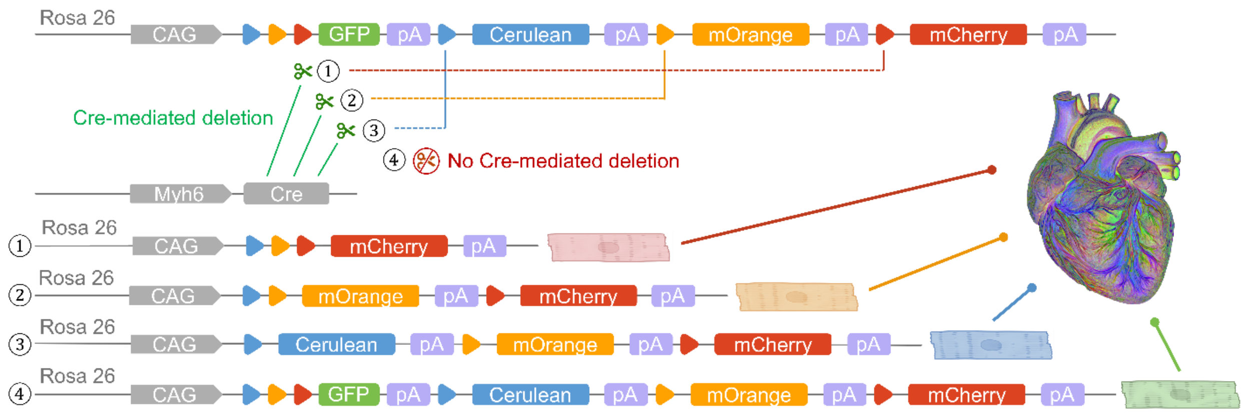

Figure S10. Development of αMHC^{Cre}; R26$^{VT2/GK}$ mouse heart. Schematic representation of the rainbow reporter system in an αMHC^{Cre}; R26$^{VT2/GK}$ mouse, illustrating the genetic modifications and expression patterns of a rainbow heart.
